# A cryptic RNA-binding domain mediates Syncrip recognition and exosomal partitioning of miRNA targets

**DOI:** 10.1038/s41467-018-03182-3

**Published:** 2018-02-26

**Authors:** Fruzsina Hobor, Andre Dallmann, Neil J. Ball, Carla Cicchini, Cecilia Battistelli, Roksana W. Ogrodowicz, Evangelos Christodoulou, Stephen R. Martin, Alfredo Castello, Marco Tripodi, Ian A. Taylor, Andres Ramos

**Affiliations:** 1 Research Department of Structural and Molecular Biology, University College London, Darwin Building, Gower Street, London, WC1E 6XA UK; 2 Macromolecular Structure Laboratory, The Francis Crick Institute, 1 Midland Road, London, NW1 1AT UK; 3 Istituto Pasteur Italia-Fondazione Cenci Bolognetti, Department of Cellular Biotechnologies and Haematology, Sapienza University of Rome, Viale Regina Elena 324, 00161 Rome, Italy; 4 Structural Biology Science Technology Platform, The Francis Crick Institute, 1 Midland Road, London, NW1 1AT UK; 5 Department of Biochemistry, University of Oxford, South Parks Road, Oxford, OX1 3QU UK; 6 Present Address: Department of Chemistry, Humboldt Universität zu Berlin, Brook-Taylor-Street 2, 12489 Berlin, Germany; 7 Present Address: School of Molecular and Cellular Biology, Faculty of Biological Sciences, University of Leeds, Woodhouse Lane, Leeds, LS2 9JT UK

## Abstract

Exosomal miRNA transfer is a mechanism for cell–cell communication that is important in the immune response, in the functioning of the nervous system and in cancer. Syncrip/hnRNPQ is a highly conserved RNA-binding protein that mediates the exosomal partition of a set of miRNAs. Here, we report that Syncrip’s amino-terminal domain, which was previously thought to mediate protein–protein interactions, is a cryptic, conserved and sequence-specific RNA-binding domain, designated NURR (N-terminal unit for RNA recognition). The NURR domain mediates the specific recognition of a short hEXO sequence defining Syncrip exosomal miRNA targets, and is coupled by a non-canonical structural element to Syncrip’s RRM domains to achieve high-affinity miRNA binding. As a consequence, Syncrip-mediated selection of the target miRNAs implies both recognition of the hEXO sequence by the NURR domain and binding of the RRM domains 5′ to this sequence. This structural arrangement enables Syncrip-mediated selection of miRNAs with different seed sequences.

## Abstract (web summary)

Syncrip is a conserved RNA-binding protein that mediates selective miRNA loading into cell-secreted exosomes, which is important for cell–cell communication. Here the authors provide mechanistic insights into how Syncrip recognises its target miRNAs by combining NMR and crystallography.

## Introduction

Exosomes are small, cell-secreted vesicles that carry specific repertoires of proteins and RNAs to recipient cells. This selective transfer of proteins and RNAs in the exosomal cargo represents an important means of inter-cellular communication^1^. Exosome-mediated microRNA (miRNA) transfer is thought to be important in various processes and systems, including the immune response^2^ and neuron-glia communication^3^. In addition, it has been implicated in a number of diseases, including cardiomyopathies^4^, neurological diseases^5^ and cancers^6^. Exosomal miRNA delivery in cancers mediates the communication between the tumour and stromal compartments. For example, it has been shown that the exosomal miRNAs in the brain microenvironment downregulate PTEN (phosphatase and tensin homologue) in nearby tumour cells^7^.

The selectivity of miRNA loading encodes the inter-cellular message carried by the exosome and a key question in the field is how this selectivity is determined at the molecular level^1^. Recent reports have shown that loading of specific miRNAs is mediated by RNA-binding proteins, four of which have been identified so far. Two of them, hnRNPA2B1^8^ (the first such protein to be identified and a close relative of hnRNPA1, a protein known to be involved in miRNA regulation^9^) and Syncrip^10^, select the target miRNAs based on the presence of short G-rich RNA sequences, which are different for the two proteins. For the other two proteins, HuR^11^, that has been linked to miRNA function before^12,13^ and YTBX1^14^, no target sequences have been identified. Importantly, hnRNPA2B1, YTBX1 and Syncrip^8,10,14^ each have multiple miRNA targets. This is consistent with a model whereby a gene regulatory signal carried by the exosome can be encoded by an ensemble of miRNA molecules that are working synergistically and that are loaded by a single regulatory RNA-binding protein. However, we have no molecular information on how these RNA-binding proteins recognise miRNA targets and mediate their exosomal localisation.

**Fig. 1:**
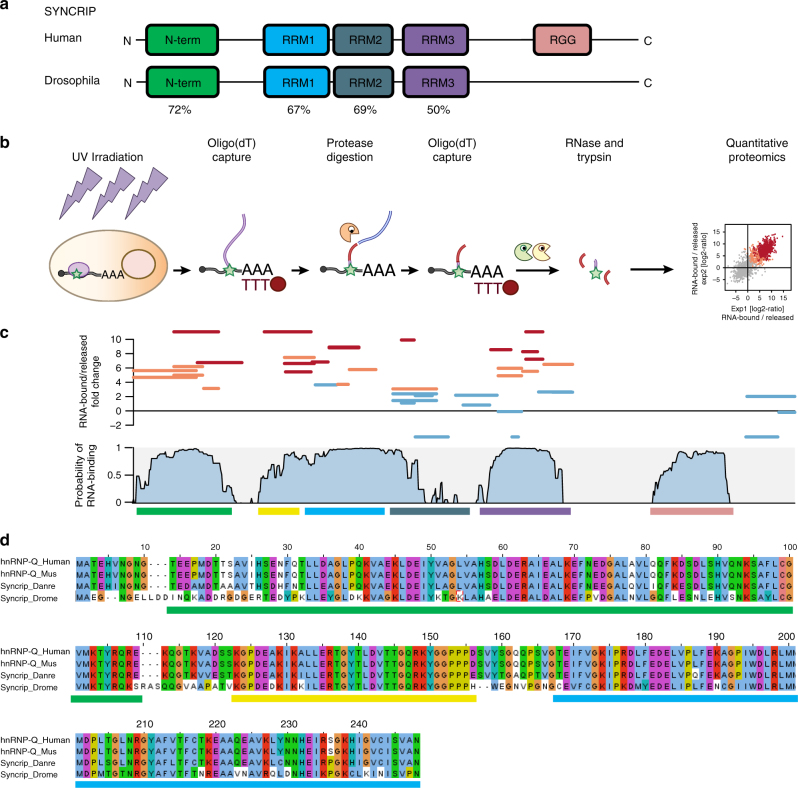
Syncrip interacts with mRNA targets using multiple RNA-binding domains. **a** Schematic of the domain organisation and sequence conservation of Syncrip protein from Drosophila and human. The domains are drawn as coloured rectangles and the sequence identity between Drosophila and human individual domains is shown below each equivalent pair. **b** Workflow of the RBDmap assay. **c** Mapping of the RNA-binding sites detected in human Syncrip by RBDmap. Top: *x*-axis represents Syncrip from N- to C-terminus. *y*-axis represent the fold change between the RNA-bound (i.e., peptides crosslinked to RNA) and released (i.e., peptides released to the supernatant after protease treatment) fractions. RNA-bound peptides identified with FDR < 0.01 are depicted as red lines. Candidate RNA-bound peptides (FDR < 0.1) are depicted in salmon. Peptides released to the supernatant after proteolytic treatment are depicted in cyan. Middle: sequence propensity to bind RNA based on the superset of RNA-bound peptides of human RBPs identified by RBDmap. This prediction was performed by shrinkage discriminant analysis employing a binary classifier, which consists of the sequences of the RNA-bound peptides (positive examples) and that of the peptides lacking RNA-binding activity (negative examples). Resulting algorithm was applied to identify the regions of Syncrip with high probability to interact with RNA. Bottom: lines indicate the different domains of Syncrip, as a reference. The colour of the line(s) corresponds to the colour of the domains in panel **a**. In addition, the conserved RNA-binding sequence N-terminal to the canonical RRM1 fold domain is indicated by a yellow line. **d** Sequence alignment of the N-terminal region of Syncrip. The coloured lines below the sequences define the boundaries of the domains as reported in **a** and **c**

Syncrip is a conserved RNA-binding protein important in neuronal and muscular development in Drosophila and mammals^15–17^. Mis-regulation or dysfunction of Syncrip is associated with severe cardiomyopathies and neuro-degenerative disorders^18–20^. In the fly embryo, Syncrip is important for the morphology and growth of the neuromuscular junction and regulates cytoplasmic vesicle-based messenger RNA (mRNA) transport^16^. In mammals, Syncrip exerts control on the length and number of neurites in mouse embryonic cortical neurons^19^ as well as the growth of nascent axons^17^, among other functions. At the molecular level, Syncrip recognises a diverse range of RNA sequences, including UACU-containing^21^ and polyA^22^ sequences, and regulates mRNA editing, transport, translation and degradation^15,21–23^. Importantly, we showed that Syncrip recognises an hEXO (GGCU/A) sequence in a set of miRNA targets and mediates their exosomal enrichment^10^. However, how Syncrip recognises its diverse ensemble of mRNA and miRNA targets is not known. Syncrip contains three conserved RRM domains (Fig. 1a), which are putative RNA-binding units, flanked by a highly conserved N-terminal domain reported to mediate the interaction with Apobec protein^24^, and a long, unstructured, less conserved C-terminus, which has been reported to mediate the interaction with synaptotagmins^25^ and a G-quartet RNA^17^. Considering the multiplicity of Syncrip RNA-binding domains and the diversity of its RNA targets, it seems plausible that several domains contribute to Syncrip’s miRNA and mRNA binding, as observed for other multi-domain RNA-binding proteins^26^.

This study explains how Syncrip’s structure and mode of RNA interaction underpins its ability to recognise a set of miRNA targets with different seed sequences to be localised onto exosomes. We identify a cryptic RNA-binding unit that recognises RNA with high-sequence specificity and show that this non-canonical RNA-binding domain is responsible for hEXO recognition in Syncrip-mediated exosomal miRNA partitioning. We also show that the domain is functionally and physically coupled by a structural element to a second RNA-binding region comprising the RRM domains, and that the coordinated interaction of multiple Syncrip RNA-binding domains is the basis of efficient recognition of the full-length miRNA. We also discuss the role this binding mode may play in the interaction with the Syncrip mRNA targets.

## Results

### Syncrip N-terminal and RRM regions interact with RNA

Data from a number of independent studies indicate that Syncrip binds to a broad range of mRNA targets by recognition of a diverse set of sequences^17,21,22,27,28^. As a first step to identifying the domains of Syncrip engaged in RNA binding in living cells, we used the RBDmap data set^29^. These data report on RNA-binding protein segments making contact with poly-adenylated RNAs within native protein–RNA complexes. They are obtained by applying ultraviolet (UV)-crosslinking to living cells, oligo d(T) capture of poly-adenylated RNAs, limited proteolysis and a second round of oligo (dT)-mediated isolation (Fig. 1b). Peptides that remain crosslinked to the RNA after this process are released by trypsin treatment and analysed by quantitative mass spectrometry. By taking advantage of this unbiased methodology we aimed to identify Syncrip’s various RNA-binding domains. As expected, seven high-confidence (FDR < 0.01) RNA-bound peptides mapped to RRMs 1, 2 and 3, suggesting that the three RRM domains function as RNA-binding domains in a cellular context (Fig. 1c, red lines and Supplementary Table 1). However, only one high-confidence RNA-binding peptide was identified in RRM2, suggesting that this domain either binds to or crosslinks with lower efficiency to RNA than the other two RRMs. Interestingly, we also noticed the presence of three high-confidence (FDR < 0.01) and one candidate (FDR < 0.1) RNA-bound peptides in a region immediately preceding the RRM1 domain (Fig. 1c, red and salmon lines, respectively), which implies additional non-canonical contacts with the RNA. Finally, we observed that two high-confidence and five candidate RNA-bound peptides mapped to the amino (N)-terminal region of Syncrip, overlapping with the ‘acidic’ domain that has previously been described as a putative protein-interaction unit. The presence of multiple high-confidence RNA-bound peptides in this region indicates it likely plays a role in the interaction with target RNAs in the cell.

RBDmap data can also be analysed at a global level to predict the likely RNA-binding regions within a protein, such as Syncrip. This analysis employs a shrinkage discriminant analysis trained with the RNA-bound peptides and the peptides lacking RNA-binding activity reported in the RBDmap data set (unpublished). Analysis of the Syncrip sequence with this algorithm revealed high-probability RNA-binding activities not only in the RRMs, but also in the ‘acidic’ N-terminal domain (Fig. 1c). This indicates that the N-terminal ‘acidic’ domain, although specific to Syncrip protein, contains patterns of short sequences often associated with RNA-binding sites in other proteins.

### Syncrip N-terminal and RRM domains are joint by an αββ motif

**Fig. 2:**
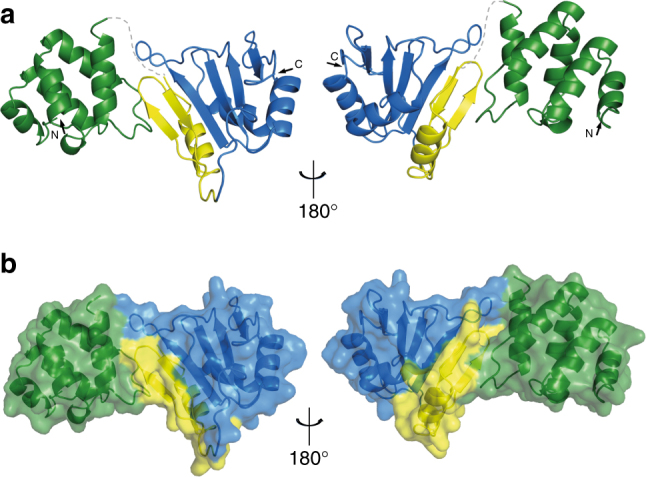
The N-terminal mRNA-binding region of Syncrip folds as a two-domain structural unit centred on a novel αββ element. **a** Cartoon representation of the structure of the NeR1 construct (aa 18–243). The N-terminal domain is coloured in green, the αββ extension in yellow and the canonical RRM1 in blue. The dashed grey line indicates the flexible linker between the two domains, which is not visible in the electron density map. **b** Surface representation of the di-domain. The RRM1 extension bridges the gap between the N-terminal and core RRM folds

In order to investigate the non-canonical N-terminal RNA-binding region of Syncrip and to provide a structural framework for our studies, we set out to determine the structure of the N-terminal region of the conserved N-terminal ‘acidic’ and RRM1 domains (NeR1 construct) (Fig. 1d and Table 1) in Human and Drosophila Syncrip proteins. The Drosophila protein crystallised in space group P2_1_ with two copies in the asymmetric unit. The 2.2 Å resolution X-ray structure (Fig. 2a) was solved by single-wavelength anomalous diffraction (SAD) using a seleno-methionine derivatised protein. The final *R*_work_/*R*_free_ are 17.4% and 20.6%, respectively, and details of the structure solution and refinement are presented in Supplementary Table 2.

**Table 1:** Syncrip constructs used in this study

Abbreviations	Construct description	Construct boundaries
N	Syncrip N-terminal domain	*Dm*: residues 16–109
		*Hs*: residues 16–109
eR1	Syncrip extended RRM1	*Dm*: residues 118–243
		*Hs*: residues 118–242
NeR1	Syncrip N-terminal and extended RRM1 domains	*Dm*: residues 16–243
		*Hs*: residues 16–242
NeR1 R60A G97L	Syncrip N-terminal and extended RRM1 domains contacting two mutated residues	*Hs*: residues 16–242
eR1R2R3	Syncrip extended RRM1, RRM2 and RRM3 domains.	*Hs*: residues 118–428
NeR1R2R3	Syncrip N-terminal, extended RRM1, RRM2 and RRM3 domains.	*Hs*: residues 16–428

**Fig. 3:**
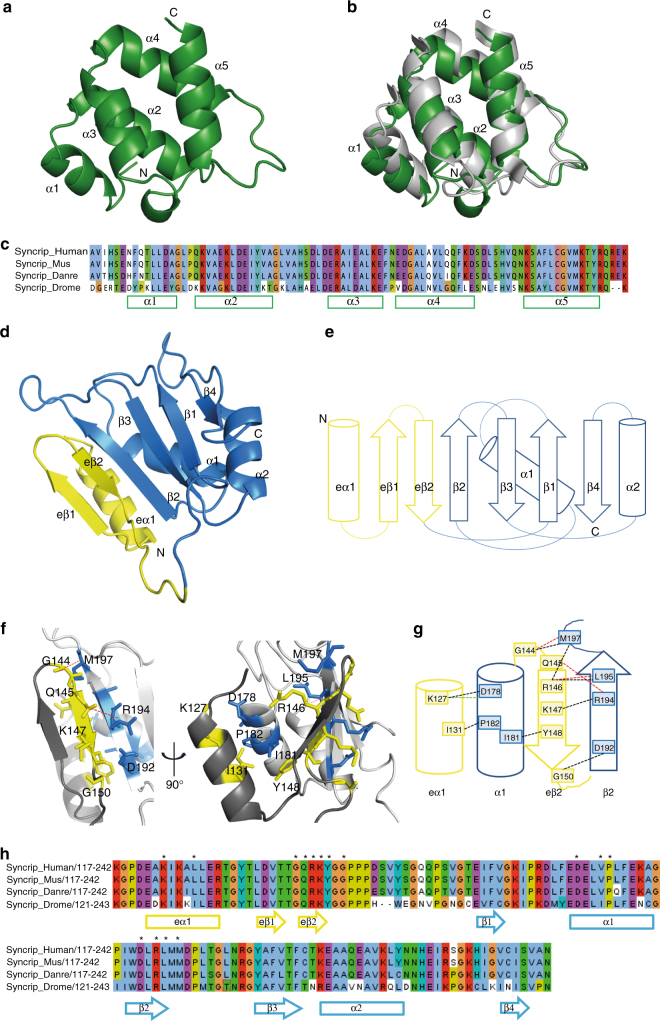
Structure of the N-terminal and extended RRM1 domains. **a** Cartoon representation of the structure of Syncrip N-terminal alpha-helical domain. The helices making up the five-helix bundle are labelled. **b** Structural superimposition of the Drosophila (green) and human (PDBID: 2MXT, grey) N-terminal ‘acidic’ domains. **c** Sequence alignment of Syncrip N-terminal domains. The secondary structure elements as observed by the crystal structure are displayed below the sequence. **d** Cartoon representation of the structure of extended RRM1. The canonical RRM fold is coloured in blue, the αββ extension in yellow. **e** Topology of the extended RRM1 domain, same colours. **f** Expanded view of the interface between the αββ extension and the canonical RRM fold. The side chains of the residues involved in the interaction are displayed as sticks, with residues in the αββ extension coloured in yellow and residues in the canonical RRM fold in blue. Hydrogen bonds are indicated with red dashed lines. **g** Schematic drawing of the contacts described in **f**. In addition to the hydrogen bonds, hydrophobic interactions and salt bridges are shown as black and green dashed lines, respectively. **h** Sequence alignment of extended RRM1. The secondary structure elements of the protein are displayed below the sequences and the residues mediating the contacts between the αββ extension and RRM1 are marked with an asterisk

The structure comprises residues G22–P243 but is missing residues 106–120, which form the linker between the N-terminal ‘acidic’ and the RRM domains and are likely disordered in the crystal. The N-terminal region is organised as a two-domain unit, with the alpha-helical N-terminal ‘acidic’ domain connected to a classical RRM fold by an αββ structural element (Fig. 2a, b and Supplementary Fig. 1a**)**. The conformation of the RRM, together with the orientation of the two domains and the inter-domain surface are essentially identical when comparing the two copies in the crystal asymmetric unit (Supplementary Fig. 1). However, the two copies show minor differences in the N-terminal ‘acidic’ domain, which has also higher temperature factors. Examination of individual domains reveals the N-terminal ‘acidic’ domain folds into a 5-helix bundle (Fig. 3a) with a hydrophobic core formed predominantly by leucine residues and a surface where 13 basic and 15 acidic residues create a network of interactions (Supplementary Fig. 2). This structure is consistent with that recently reported for the isolated N-terminal ‘acidic’ domain in the human protein^30^, although some differences exist most noticeably in the position of helix-3 (Fig. 3b). This is not surprising considering the sequence conservation of the domain (Fig. 3c).

Examination of the two-domain structure also reveals that the RRM1 domain is flanked by a N-terminal αββ module (Fig. 2a, b and 3d, e) to create an extended RRM (eRRM) fold. The structure of this extended fold shows that packing between the extension and the RRM is mediated mainly by hydrophobic contacts between the eβ2 and β2 strands and between the eα1 and α1 helices with a small number of hydrogen bonds and one salt bridge also contributing (Fig. 3f, g). Within the interface, the hydrophobic side chains of the amino acids of the extension and core domain are inter-digitated and their packing buries a large (1640 Å^2^) solvent-accessible surface. This likely explains our observation that the RRM1 domain is not expressed in a soluble form in *E. coli*. Finally, inspection of a sequence alignment of Syncrip family members also reveals a large degree of sequence conservation in this region of the proteins further supporting the idea that the αββ extension is a defining feature of the Syncrip family (Fig. 3h).

**Fig. 4:**
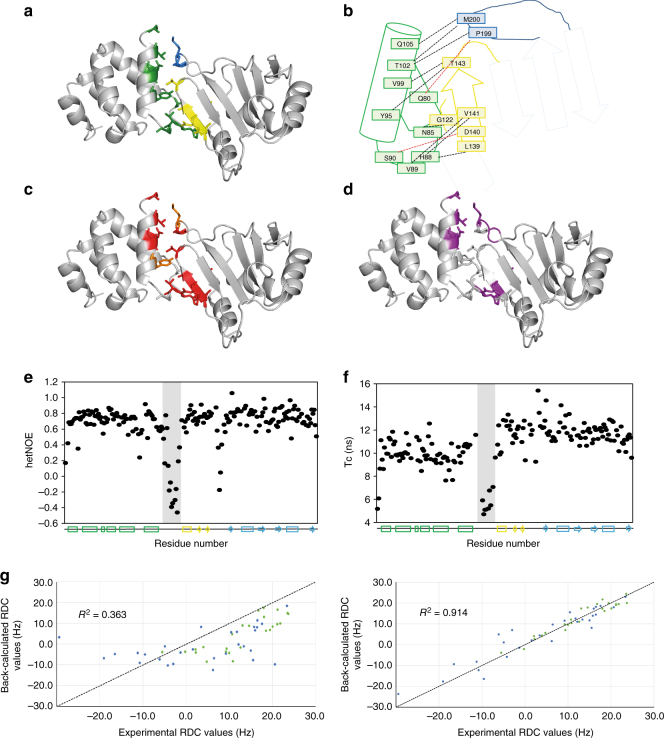
Syncrip N-terminal and extended RRM1 domains are coupled by a specific and dynamic interface. **a** Cartoon representation of the structure of Syncrip N-terminal region (NeR1). The side chains of the amino acids mediating the inter-domain interaction are displayed as sticks and coloured in green (N-terminal domain), yellow (αββ extension) and blue (RRM core domain). **b** Schematic of the inter-domain contacts using the same colour coding as in panel **a**. Dashed lines represent the interactions between residues. Hydrophobic interactions are in black, hydrogen bonds in red. **c** Conservation of the inter-domain interface. Residues conserved in Drosophila and human are coloured in red (identical) or orange (same residue type). **d** Amide resonances of either domain that change in the context of two-domain construct are coloured in purple on the NeR1 structure. The changes observed are limited to the interaction surface observed in the crystal, indicating that the conformation observed in the X-ray structure represents the major conformer in solution. **e** Heteronuclear NOE and **f** rotational correlation time (*τ*_c_) values mapped along the protein’s sequence. The secondary structure is drawn at the bottom of each panel. The trend of NOE and *τ*_c_ values along the sequence indicates that two domains are stably folded while amino acids in the inter-domain linker (grey background) and in the loop between the canonical fold and αββ extension of the RRM are more flexible. The different *τ*_c_ values indicates that the two domains do not tumble together. **g** Correlation between experimentally measured RDCs, and RDCs back-calculated from the crystal structure using the programme Module. In the left-hand panel a single tensor was used to fit the data, while in the right-hand panel two tensors corresponding to the two domains (green: N-terminal domain blue: extended RRM1) were used in the fit. The two-tensor fit shows a much better correlation with the data (the correlation coefficient between measured and calculated RDCs was 0.914 instead of 0.363) confirming that the two domains do not behave as a single stable structural unit in solution

The structure revealed that the interaction between the N-terminal ‘acidic’ domain and the eRRM1 is mediated largely by the last helix of the N-terminal domain (α5) making contacts with the αββ RRM-extension, and specifically with residues of the first β-strand (eβ1). Additionally, residues of the core RRM β1-β2 loop also make contact with the N-terminal ‘acidic’ domain (Fig. 4a, b). The domain–domain contacts are largely hydrophobic, but do include two hydrogen bonds (Fig. 4b). The residues involved in these interactions are conserved from Drosophila to humans (Fig. 4c) which, considering that the individual domains are stable and soluble in solution, indicates that the contacts are functionally important. However, in contrast to what was observed for the interaction between the αββ element and the core RRM1, this interface is not tightly packed and the interaction buries only 699 Å^2^ of solvent-accessible surface.

We wondered whether, in solution, the two domains interact in the same orientation as that observed in the crystal, and whether this interaction represents a rigid and unique conformation. In order to address these points, we first used ^15^N-correlation nuclear magnetic resonance (NMR) spectroscopy to assess differences in the amide resonances (i.e., the chemical shift perturbation, CSP) of the two domains in isolation (N and eR1 constructs) (Table 1) and in the context of the two-domain construct. A comparison of the spectra showed that significant changes were only found in residues that are part of the interaction surface in the X-ray structure, indicating that a conformer with this surface of interaction is highly populated in solution and it is likely that an equivalent inter-domain interaction is taking place in solution and in the crystal (Fig. 4d). Next, we assessed whether the two domains are locked together in a unique orientation. First, we used ^1^H-^15^N heteronuclear NOEs to evaluate which regions of the di-domain were the most flexible. The NOE values (Fig. 4e) indicated that the two linkers joining the αββ unit to the N-terminal domain and the core RRM are undergoing significant high-frequency motions. Indeed, these linkers do not participate in the inter-domain interactions observed in the crystal structure. We then determined the rotational correlation time (*τ*_c_) of each of the domains within the two-domain construct (Fig. 4f). Surprisingly, we observed a significant difference in *τ*_c_ (*τ*_c_ of the N-terminal domain is 10.0 ± 0.9 ns, *τ*_c_ of the extended RRM1 domain is 11.8 ± 1.0 ns) that indicates that the two domains are not tightly coupled. The dynamic nature of the relationship between the domains was confirmed using a second NMR observable. We measured the ^15^N amide residual dipolar couplings (RDCs) for the two-domain construct and compared the experimentally measured RDC values to the RDC values back-calculated from the crystal structure using either a single tensor for the two domains or two independent tensors, one per domain; the latter representing a molecule where the domains are not tightly coupled. This comparison (Fig. 4g, h) shows that the RDC set calculated from a two-tensor fitting, but not the one calculated from a one-tensor fitting, correlates well with the experimentally measured set. The RDC data indicate that the structure of the individual domains is the same in solution and in the crystal, but also that the domains are not locked together in a unique orientation. To further validate that the conformation observed in the X-ray structure is compatible with the solution data we carried out a data-driven molecular docking protocol with HADDOCK^31,32^ employing the CSP and RDC data. The resulting models cluster in two main families (see Materials and methods), with the largest, lower energy family superimposing closely with the crystal structure (Supplementary Fig. 2), while in the second smaller family, the surface of interaction is very similar but the orientation of the two domains is 180-degree rotated. Notably, a single set of RDC data does allow for symmetric positioning of the NH vectors, which would explain the inter-domain orientation in this second family and so it seems likely this second family is an artefact that reflects the limits of the solution data. Altogether, the HADDOCK modelling and solution data indicate that the X-ray structure represents a highly populated conformer in solution and that the interaction between the two domains is specific but dynamic with the domains spending a non-negligible part of the time detached. An important question is how the coupling we observe between the two domains is linked to protein function and target recognition.

### Syncrip N-terminal domain is a conserved RNA-binding domain

**Fig. 5:**
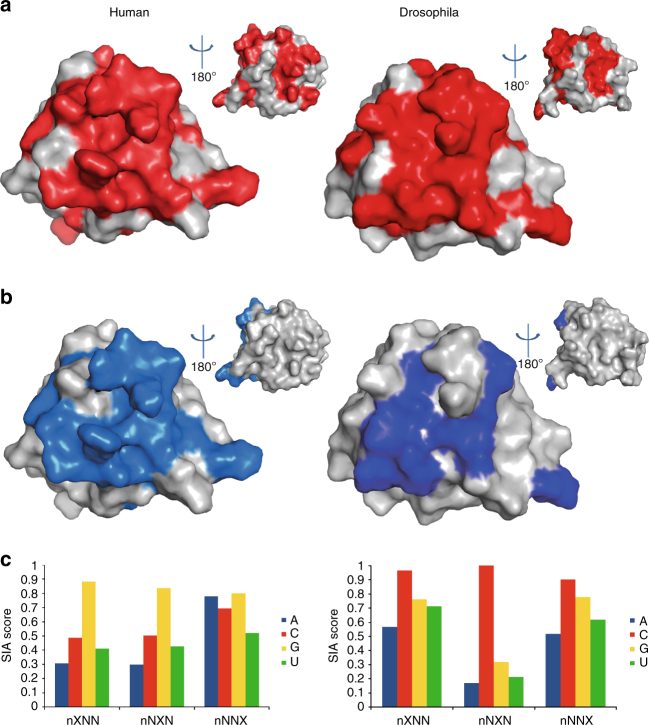
The N-terminal domain of Syncrip is a novel sequence-specific N-terminal unit for RNA recognition (NURR). **a** Residues conserved (identical) in human and Drosophila are coloured in red on the structures of human (PDB ID: 2MXT) and Drosophila Syncrip (this study) N-terminal domains. The smaller images show 180° rotations of the structures. **b** Chemical shift changes upon binding the randomised 4N RNA are mapped in blue on the surface of the NURR domains. The smaller images show 180° rotations of the structures. **c** SIA scores for the human and Drosophila NURR domain, reflecting the nucleobase preferences at the different positions of the bound sequence. In this representation, the relative height of the four columns in each position represent the relative preference of the domain for the four nucleobases

The N-terminal ‘acidic’ domain of Syncrip is conserved throughout evolution (Fig. 1c), although it has no close structural homologue outside the Syncrip family. The domain has been reported to be essential for the interaction with the cytosine deaminase APOBEC1^33^ and, also based on a predicted overall strongly negative charge, it was proposed to act as a protein–protein interaction module. However, our structural analysis shows that many of the negatively charged residues predicted to be part of the domain are in fact located in the neighbouring unfolded regions and rather being than strongly acidic, the domain actually has an overall charge that is close to neutral. In order to obtain some initial information on the determinants of the N-terminal domain’s functional interactions, we mapped the conserved residues on the domain structure (Fig. 5a). These data revealed a large conserved surface likely to be important for the interaction with its functional partners. This surface is centred on a patch of positive and hydrophobic amino acids (Fig. 5a and Supplementary Fig. 3) and is, therefore, a good candidate to mediate the mRNA interaction identified in the mRNA capture assays. This was tested using NMR spectroscopy, initially on a construct including the N-terminal domain only (construct N). As the RNA sequence recognised by the domain was unknown, RNA binding was initially investigated by titrating short randomised oligonucleotides of increasing length (4 and 5 nucleotides that we name 4N RNA and 5N RNA) into the protein and monitoring the binding using the CSP observed in ^15^N-correlation fingerprint NMR experiments. Titration with the different RNA oligos changes the position of selective amide resonances from residues in two of the five helices (Helices 3 and 5) that are part of the domain’s conserved surface (Fig. 5b). Further, this RNA-binding surface is unchanged in titration with 4N RNA and 5N RNA.

Next, we wanted to determine whether RNA recognition by the domain, which we designated NURR, is specific and whether this specificity is conserved during evolution. In order to define the sequence specificity, we performed scaffold independent analysis (SIA)^34^ on both Drosophila and human Syncrip. SIA examines the sequence specificity of RNA-binding domains mediating moderate affinity interactions. The method is based on the comparative analysis of the binding of a protein to quasi-degenerate sets of RNA pools using NMR spectroscopy. For each position of an RNA sequence to be examined four pools are used where a single position is occupied by either an A, U, C, or G while the other positions are occupied by a randomised mix of the four bases. NMR is used to assess the binding of the four pools to the protein in a comparative fashion and the comparative SIA scores^34^ reported (Fig. 5c) represent the relative preference for a base with respect to the others in a specific position. Our SIA assays delivered an unbiased report of the nucleobase preferences of the domain in human and Drosophila (Fig. 5c). These SIA data revealed that, in Drosophila, the NURR domain recognises a C in the bound sequence at position 2 with very high specificity, while the nucleotides in positions 1 and 3 are bound with lower specificity (Fig. 5c). Importantly, and in contrast to the Drosophila protein, human Syncrip recognised a G in both the first and second bound position (Fig. 5c) and discriminates against U in the third bound position, which has much lower specificity. These data suggest that the human and Drosophila proteins bind RNA with the same surface but interact with different sets of targets.

**Fig. 6:**
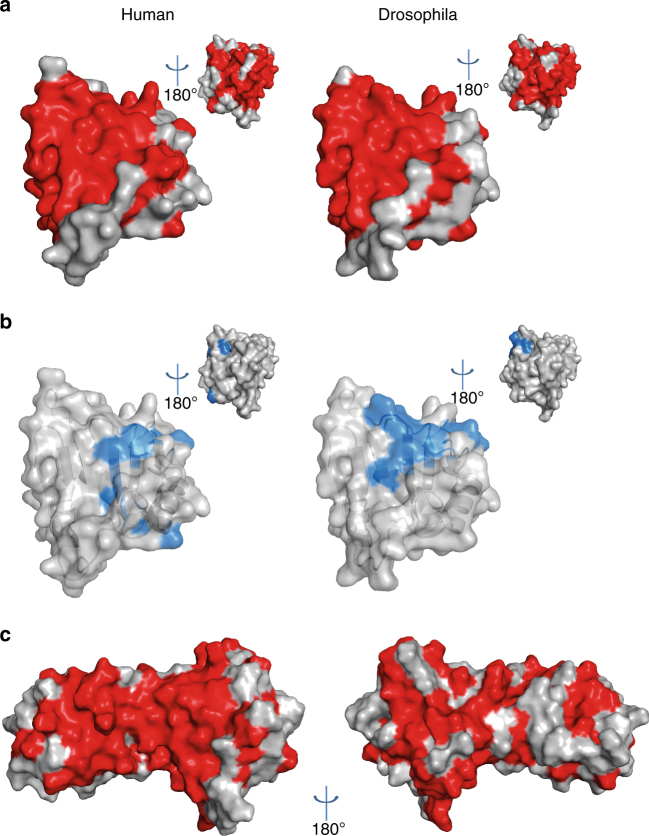
Syncrip extended RRM1 domain binds RNA using the canonical RRM β-sheet surface. **a** Residues conserved (identical) in human and Drosophila are coloured in red on the Drosophila structure (this study) and a derived human protein structural model (Swiss-model) of the extended RRM1 domain. The smaller images show 180° rotations of the structures. **b** Chemical shift changes upon binding of quasi-randomised RNA oligos (see materials and methods) are mapped in blue on the surface of extended RRM1 domain in human and Drosophila. The small images show 180° rotations of the structures. **c** The interaction of the NURR and extended RRM1 domains aligns the conserved (RNA binding) surfaces of the two domains. Conserved residues are coloured in red on the structure of the two-domain, NeR1 construct

To complement this analysis, we explored RNA binding by the eRRM domain (construct eR1), where the canonical RRM β-sheet RNA-binding surface is extended by the two β-strands of the αββ element. This surface is highly conserved (Figs. 1d and 6a), and includes the aromatic residues in the RRM’s RNP1 and RNP2 motifs associated with canonical RNA binding. Chemical shift changes in ^15^N-correlation NMR spectra recorded with different RNA pools confirmed that this conserved surface mediates RNA binding (Fig. 6b), although the fast exchange regime and relatively small chemical shifts observed suggests the interaction made by the isolated eRRM1 domain is not of high affinity.

Notably, in the crystal structure the conserved RNA-binding surfaces of the RRM and NURR domain are aligned by the physical coupling between the two domains (Fig. 6c), providing a first rationale for this coupling. This creates a single di-domain surface for RNA recognition where a canonical RRM-RNA binding mode positions the RNA in a 3′ (NURR-bound) to 5′ (RRM-bound) orientation. The RRM domains of Syncrip are joined by very short linkers and, arguably, the αββ extension of RRM1 connects RNA binding by the NURR domain to that of the entire RRM region of the protein.

### NURR domain–hEXO RNA interaction mediates miRNA recognition

Our recent work has shown that Syncrip recognises a GGCU/A (hEXO) sequence present in a group of miRNAs, and that this interaction directs the loading of these miRNAs into exosomes^10^. Indeed inserting this sequence in a non-target miRNA (miR29a-1) leads to its exosomal loading^10^. Our present study shows that the human NURR domain recognises, with high specificity, a GG sequence that matches the core nucleotides of the hEXO sequence (Fig. 5c), and we hypothesise that the NURR–hEXO interaction provides a key element for Syncrip-miRNA recognition.

**Fig. 7:**
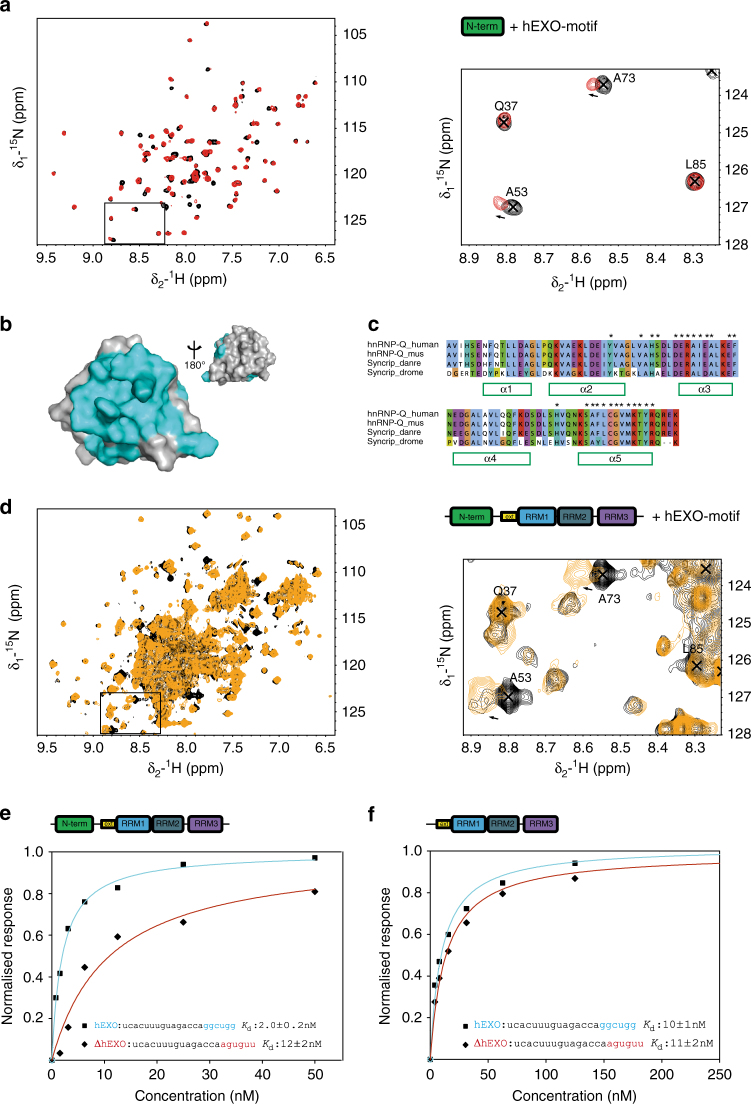
The NURR domain mediates the specific recognition of miR-3470 hEXO-motif. **a** Binding of the NURR domain to the AGGCU hEXO-motif monitored by NMR. Left: superimposition of the free (black) and RNA-bound (1:1 ratio, red) protein spectra. Right: zoom of the boxed region. Residues names are annotated. **b** Residues whose amide resonances are affected by the binding of the hEXO RNA are mapped in blue on the surface of the NURR domain. **c** Residues with significantly perturbed resonances upon hEXO binding are indicated with an asterisk above the sequences of the human and Drosophila proteins. **d** Binding of the four-domain NeR1R2R3 construct to the AGGCU hEXO-motif. Left: superimposition of the free (black) and RNA-bound (1:1 ratio, orange) protein spectra. Right: zoom of the boxed region. Residues names are annotated as in **b**. **e** Binding of the four-domain (NeR1R2R3) protein to wild-type miR-3470 (hEXO) and mutant miR-3470 (ΔhEXO) monitored by BLI. The hEXO and mutated sequences are highlighted in the inset in blue and red, respectively. BLI response values are plotted against the protein concentrations and normalised for better comparison. Dissociation constants are reported. **f** Binding of the three-domain construct (eR1R2R3) lacking the NURR domain to wild-type and mutant miR-3470, as in **e**. In the absence of the NURR domain, the protein no longer recognises the cognate RNA

To test our hypothesis, we first examined the NURR–hEXO interaction in isolation using NMR and confirmed that the short hEXO RNA binds to the conserved RNA-binding surface of the domain by monitoring chemical shift changes during a titration of the protein with the RNA and mapping the perturbed resonances on the domain structure (Fig. 7a–c Supplementary Fig. 4). Then we performed an equivalent titration using a 4-domain hSyncrip construct containing the NURR domain plus the three RRMs (NeR1R2R3). Although the spectral quality of the larger protein is, as expected, lower, the comparison of the two titrations showed that none of the distinguishable resonances of the three RRMs changed upon RNA addition (Fig. 7a, d and Supplementary Fig. 5, see also the later comparison with the RRM resonance shifts obtained by binding a full-length miRNA). Instead, equivalent changes are observed in the amide resonances of the NURR domain upon RNA binding in isolation and within the larger protein (Fig. 7a, d and Supplementary Fig. 5). This indicates that the RNA selects specifically the NURR domain over the three RRM domains and the features of the interaction do not change in the context of the four-domain protein, confirming the importance of the specific NURR–hEXO RNA recognition (Fig. 7a, d).

In order to establish a direct link between the NURR domain and Syncrip recognition of the hEXO sequence within a full-length miRNA target we used biolayer interferometry (BLI) assays. As a model we chose miR-3470, the miRNA that (i) is most efficiently pulled down by Syncrip after crosslinking and (ii) shows the strongest change in exosomal partitioning upon silencing Syncrip^10^. First, we compared the binding of the four-domain RNA-binding region of Syncrip (NeR1R2R3) to wild-type full-length miR-3470 and to miR-3470 with a mutated hEXO sequence, and showed that mutation of the hEXO sequence decreases the binding affinity ~6-fold (Fig. 7e). This experiment established that our in vitro assays can discriminate between cognate and non-cognate targets. Then, we showed that recognition of the hEXO sequence is dependent on the presence of the NURR domain, as the eR1R2R3 construct, that does not contain the NURR domain, can no longer discriminate between wild-type and mutated miR-3470 in a BLI titration of the RNA with the three-domain construct (Fig. 7f). It is worth mentioning that mutation of the hEXO sequence and removal of the NURR domain lead to the same decrease of affinity.

**Fig. 8:**
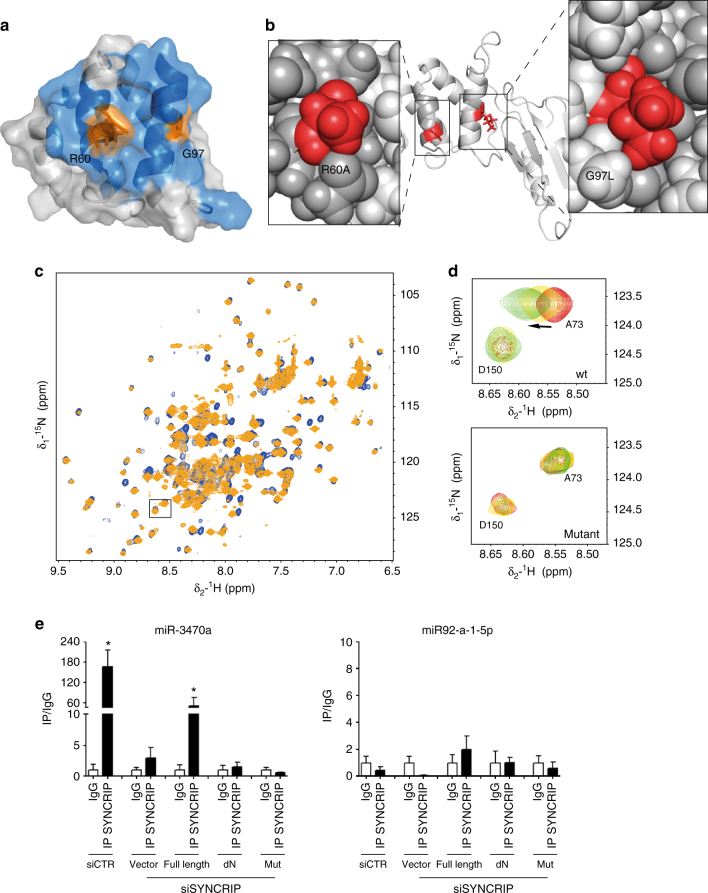
Eliminating the RNA-binding activity of the NURR domain impairs Syncrip interaction with miR-3470 in the cell. **a** Surface representation of human Syncrip N-terminal domain, residues affected by RNA binding are coloured blue and the two mutated residues coloured orange. **b** Model of Syncrip NeR1 R60A G97L mutant. The side chains of residues A60 and L97 are coloured red. The central image shows a cartoon representation of the protein, the two side panels show the side chains of residues close to the mutations in space filling representation. The model was built using SwissMode, using the structure of the Drosophila NeR1 two-domain protein as template, as the G97 residue is close to the N-eR1 interface. **c** The R60A G97L double mutation does not change the structure of the protein but impairs RNA binding. (Left) Superimposition of the ^15^N-correlation spectra of the wild-type (blue) and mutant (orange) NeR1 protein, showing that only minor changes are observed upon mutation and that the structure of the protein is conserved. **d** The R60A G97L double mutation eliminates RNA binding by the N-terminal domain. The changes in a representative peak of the wild-type (top) and mutant (bottom) proteins upon addition of a 1:1 and 1:2 ratio of hEXO RNA targets are displayed in this spectral superimposition. Peaks in the RNA-binding surface change upon titration of the RNA into the wild-type, but not the mutant protein. **e** RIP assays with anti-SYNCRIP antibody and, as control, preimmune IgG, on lysates from hepatocytes knocked-down for endogenous Syncrip by short-hairpin RNA (shRNAs) approach (shSyncrip). A scrambled shRNA was used as control (shCTR) as previously reported^10^. The cells were transduced with either an empty vector (Vector) or vectors overexpressing human WT Syncrip (full length), human ΔNURR (dNURR) and human mutant Syncrip (R60A-G97L). miRNA 3470a (left) and miRNA 92a-1-5p (right) (hEXO miRNA and non-hEXO miRNA, respectively) levels in immune-precipitated samples were determined by RT-qPCR and reported as IP/IgG. Data are means ± SEM of three independent experiments. Statistical significance was determined by parametric paired *t*-test analysis (*p < 0.05)

Finally, we tested whether RNA binding by the NURR domain affects the specific recognition of the RNA targets in the cellular environment. To probe this, we used both a protein construct where the NURR domain had been deleted (ΔNURR) and a variant of the domain where RNA binding has been eliminated through the introduction of two point mutations that do not change the protein structure. These structure-driven mutations changed a positively charged side chain in the RNA-binding surface to alanine (R60A) and introduced a bulky hydrophobic side-chain to disrupt protein-RNA packing (G97L) **(**Fig. 8a**)**. Modelling of these changes on the protein structure indicated that the mutated side chains are unlikely to interfere with the protein fold (Fig. 8b), and we tested both the RNA-binding function and structure of the double mutant using NMR spectroscopy. For these assays, we used the two-domain construct as the G97 residue is close to the interface between the NURR and extended RMM1 domains. Superimposition of the NMR spectra of the NeR1 construct, wild-type and mutated confirmed that the double substitution does not change the structure or destabilise the protein. Titration of the mutant with the hEXO sequence confirmed that the double mutation impairs the interaction with the RNA at 50 µM concentration and 1:4 protein: RNA ratio (Fig. 8c). Then, the selective binding capacity in the cell of human WT Syncrip was compared to that of the NURR domain deletion and R60A/G97L mutants using RNA-immunoprecipitation (RIP) after UV-crosslinking, that reports directly on protein–RNA interactions^35^. RIP analysis (as in Santangelo et al.^10^) of miR-3470 and the negative control non-hEXO miR-92a-1-p was performed using extracts of cells silenced for endogenous Syncrip expression after introduction of WT Syncrip, Syncrip ΔNURR or Syncrip R60A G97L by retroviral transduction. As shown in Fig. 8d, e the capacity of both mutants to immuno-precipitate the hEXO-containing miR-3470 is impaired with respect to the WT full-length protein, confirming the key role of the NURR domain in the recognition of the miRNA targets.

### The NURR–RRM coupling is important for RNA target selection

Our previously published cellular assays identified the hEXO sequence as the only sequence required for exosomal miRNA partitioning. However, the structural work we present here indicates that RRM and NURR binding surfaces are coupled and that this coupling is likely to play some role in the interaction. One question is whether this role is exclusively to increase the affinity of the interaction or whether binding of the RRM domains may contribute to selectivity in a non-sequence-dependent fashion, i.e., by providing some previously hidden structural restraint. To de-convolute the two possibilities we have obtained a mechanistic model for protein–RNA recognition.

**Fig. 9:**
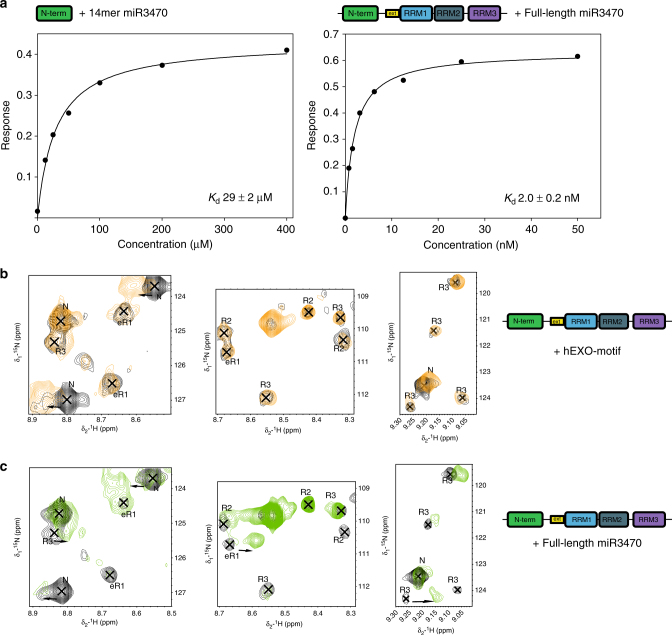
The three RRM domains of Syncrip participate in and are required for the specific high-affinity interaction between Syncrip and miR-3470. **a** Binding of the human Syncrip N and NeR1R2R3 proteins to a 14mer miR-3470 RNA (left) and to a full-length miR-3470 (right), monitored by BLI. The BLI response values are plotted against protein concentration, fitted isotherms are shown as continuous lines. **b** Expanded view of three well dispersed regions of the HSQC spectrum of human Syncrip NeR1R2R3. The free spectrum (black) is superimposed with that of the protein bound to the AGGCU hEXO-motif at 1:1 ratio (orange). Upon addition of the RNA, resonances belonging to the NURR domain RNA-binding surface shift, while resonances of the RRM domains are not perturbed. Peaks are labelled according to the domain the residue locates to. **c** Expanded view of HSQC spectrum as in **b**, but employing full-length miR-3470. Upon addition of the RNA, resonances from all four domains are perturbed demonstrating their involvement in binding

As first step, we assessed the contributions of the NURR and RRM domains to overall affinity. Our BLI and NMR data so far show that the NURR domain interacts with the isolated hEXO sequence with a weak-to-intermediate affinity and we wanted to test whether this applies also in the context of a longer RNA target. Using BLI, we measured a 29 µM* K*_d_ for the interaction between the NURR domain and a miR-3470-derived 14-nucleotide RNA (Fig. 9a and Supplementary Fig. 6), confirming that the domain binds to the RNA target with moderate affinity. By contrast, the four-domain NeR1R2R3 protein binds the full-length miR-3470 with very high affinity (*K*_d_ ~ 2.0 nM) (Figs. 7e and 9a). The four orders of magnitude increase in binding affinity indicates that the RRM domains are essential for a high-affinity interaction. Further, the physical coupling between the NURR and eRRM1 domains determines the direction of binding of these domains along the RNA molecule, positioning the NURR domain 3′ to the RRMs. All-together data show that the NURR and RRM domains cooperate to achieve a selective, high-affinity Syncrip-miR-3470 interaction, implying that the RRM domains must bind 5′ of the hEXO sequence, which is here located at the 3′ end of the miRNA. We then titrated the four-domain protein with the full-length miR-3470 and monitored the binding using ^15^N-correlation NMR spectroscopy. The NMR spectra show that the interaction between the RRM region and the miRNA involves both RRM1, RRM2, and RRM3 (Fig. 9b, c). We discuss below how the cooperation and positioning of the NURR and RRM units on the miRNA targets is related to the selection of a set of hEXO miRNAs with different seed sequences in the cell.

## Discussion

The loading of selected miRNAs into exosomes is a recently discovered mechanism for cell–cell communication that is important to the development and function of a broad range of tissues. Selectivity in miRNA loading is mediated by RNA-binding proteins, which recognise specific sequences on the target miRNAs. Here, we have focussed on the Syncrip protein to investigate the molecular basis of the selection of its miRNA targets. Syncrip is an abundant and conserved RNA-binding protein that binds both mRNA and miRNA targets and regulates mRNA transport, editing and translation degradation^15,21–23^, as well as miRNA partitioning in the exosomes^10^. However, to date we do not have a quantitative and mechanistic understanding of how RNA is targeted and regulated by Syncrip. This understanding is crucial to gain insight into the mechanistic basis of Syncrip-mediated exosomal partitioning of miRNAs.

The output of our RBD analysis indicates that the three RRM domains in the central region of Syncrip cross-link to RNA in vivo. Unexpectedly, the N-terminus of Syncrip, which includes an N-terminal domain and a conserved sequence N-terminal to RRM1, also interacts with RNA. As a first step to understanding Syncrip-RNA binding at the molecular level, we determined the crystal structure of this non-canonical RNA-binding region. The structure shows that the N-terminal and RRM1 domains are brought together by an αββ structural element created by the folding of the highly conserved sequence amino-terminal to RRM1. One question is whether and how this coupling is functionally relevant. The αββ motif aligns the two highly conserved RNA-binding surfaces of RRM1 and NURR to create one continuous RNA-binding surface and to establish the direction of how the NURR and RRM(s) position on the RNA target, providing a first indication that the physical coupling between domains is likely to be important to Syncrip RNA binding.

Unexpectedly, our work shows that the N-terminal NURR domain of Syncrip is a RNA-binding domain with a highly conserved RNA-binding surface. Further, our SIA assays and titration analyses show that RNA binding by the NURR domain is sequence specific, consistent with a direct role in the selection of Syncrip target RNA target sequence(s). Indeed the NURR domain binds to the hEXO-motif (GGCU), whose recognition by Syncrip mediates exosomal partitioning^10^. BLI data confirm that Syncrip specifically recognises the hEXO sequence within the miR-3470 target and that the removal of the NURR domain impairs this Syncrip-hEXO recognition. These assays directly relate hEXO-mediated miR-3470 recognition to the hEXO–NURR interaction, explaining the molecular basis of the reported miRNA target selectivity. This result was tested to the cellular environment with the design of the double mutant where the RNA-binding function of the protein is selectively impaired. When tested using RIP assays abolition of NURR RNA-binding function also resulted in a loss of miR-3470 pulldown confirming that RNA binding by the NURR domain is essential for target recognition in the cell. It is worth highlighting that the sixfold effect on binding affinity we observe in our in vitro two-component system translates into a loss of binding in our cellular assays. This emphasises how relatively modest changes in the affinity for an RNA target may result in drastic changes in the protein–RNA association in the cell, where other proteins and RNAs are competing for interacting partners.

A second question important to understanding Syncrip-miRNA recognition is whether the NURR domain is a stand-alone high-affinity, high-specificity miRNA recognition element that mediates the interaction with the miRNA targets, or whether and how the RRM domains contribute to the interaction. We show that, in isolation, the NURR domain binds to the miRNA target with moderate affinity (~29 µM *K*_d_), while high-affinity binding (~2.0 nM* K*_d_) to the target miRNA requires the additional interaction of the RRM domains. That is, the NURR and RRM domains act together to achieve a specific and high-affinity interaction with the Syncrip RNA targets. Importantly, the contacts between the NURR and eRRM1 domains position the RRM domains 5′ of the NURR domain on the bound RNA sequence. This provides a previously hidden structural constraint and implies that for Syncrip to interact with a target miRNA with high-affinity and specificity the hEXO sequence cannot be positioned at the 5′ end of the miRNA. The 5′ end of a miRNA molecule is occupied by the seed sequence, which is the main determinant of target recognition in canonical miRNA–mRNA target interaction, while sequences 3′ of the seed are less functionally constrained. Arguably, this mutational freedom would facilitate establishing a regulatory sequence (the hEXO sequence) common to a set of miRNAs with different targets. Therefore, we revisit the rules of recognition previously drawn and that propose the architecture of the RNA-binding region of Syncrip underpins its ability to recognise a common sequence in miRNAs with very different seed sequences and to efficiently regulate a group of miRNAs with very different target specificities.

It is worth mentioning that, in addition to recognising the hEXO sequence, the NURR domain makes contact with mRNAs in our mRNA interactome pull down experiments^29^. Interestingly, in Drosophila the domain recognises a C with high specificity in a central position of the sequence (Fig. 5c). The different specificity observed for the human and Drosophila proteins indicates that the highly conserved N-terminal domain of Syncrip has maintained its RNA interaction surface but has dramatically changed its sequence specificity during evolution, arguably to adapt to different sets of RNA targets. An open question, also relevant to a number of other multi-domain RNA-binding proteins, is how the different classes of RNA targets have contributed to define Syncrip’s structure and sequence specificity. As a first step, when the RNA interactome of the two proteins becomes available it would be interesting to assess how differences in the RNA target sequences of the human and Drosophila Syncrip relate to changes in the specificity of the different domains of the protein, also relate these differences to structure using recent methods for unbiased structural analysis of RNA-binding proteins^36^.

This work examines the molecular basis of target selection in Syncrip-mediated exosomal miRNA loading and finds that recognition of the miRNA targets is, unexpectedly, mediated by the cooperation between a NURR RNA-binding domain and three RRM domains. In the case of hnRNPA2B1, the first protein to be reported to mediate exosomal partitioning of selected miRNAs target selection depends on the recognition of a short sequence found mainly in the 3′ end of the miRNA^8^, and the protein contains two RNA-binding RRM domains. We expect future work will explore whether the Syncrip and hnRNPA2B1 proteins adopt a similar strategy of miRNA recognition, and future work will investigate how the high-affinity interaction between Syncrip and the target RNA is regulated.

## Methods

### Plasmids for protein production

The genes encoding *D. melanogaster* Syncrip isoform F (Uniprot A4V364) and codon-optimised *H. sapiens* Syncrip/hnRNP-Q (UniProt O60506, DNA purchased from GenScript) (Supplementary Table 3) were inserted into pET-52 SUMO or pET-5247 using Ligation Independent Cloning (LIC) to produce an HRV 3C cleavable amino-terminal His_6_SUMO and Hisfusion as described previously in ref.^37^. For in vitro studies, the hSyncrip NeR1 R60A G97L double mutant protein was produced using the Quikchange Site Directed Mutagenesis Kit (Agilent) and the wild-type protein expression vector as template. Mutants of the full-length hSyncrip for eukaryotic expression were prepared using the Quickchange protocol (primers sequences in Supplementary Table 4) employing the retroviral pcLBX plasmid (as described in Santangelo et al.^10^) expressing the wild-type gene as template. The sequences of all constructs were confirmed by DNA sequencing prior to expression.

### Protein expression and purification

The human and Drosophila Syncrip proteins were expressed in the *E. coli* strain BL21 (DE3) Gold (Agilent) and purified using the same protocol. Bacterial cultures were grown at 37 °C to a density of OD_600_ _=_ 0.5–0.8 and protein expression then induced by addition of 0.5 mM isopropyl-β-d- thiogalactoside (IPTG). Prior to induction, cultures were cooled and maintained at 20 °C for protein expression overnight. Cell pellets were harvested by centrifugation for 7 min at 8600 × *g* and then resuspended in lysis buffer (10 mM Tris-HCl pH 8, 1 M NaCl, 2 mM β-mercaptoethanol) containing (1 mg mL^−1^ lysozyme, 10 mg mL^−1^ of DNase1, 1 tablet per 50 mL protease inhibitor tablets (Complete Mini, Roche), and 1 µL mL^−1^ of Triton X-100) and lysed by sonication. The cell lysate was then centrifuged for 45 min at 23,600 × *g*, the supernatant was collected and applied onto a Ni-Sepharose resin column (bed size 3 mL per litre culture). The column was washed with 40 mL wash buffer I (10 mM Tris-HCl, 10 mM Imidazole, 1 M NaCl, 2 mM β-mercaptoethanol, pH 8) and 20 mL wash buffer II (10 mM Tris-HCl, 30 mM imidazole, 1 M NaCl, 2 mM β-mercaptoethanol, pH 8). Bound proteins were eluted with 40 mL of elution buffer (10 mM Tris-HCl, 300 mM Imidazole, 1 M NaCl, 2 mM β-mercaptoethanol, pH 8). Subsequently 350 µL of human rhinovirus 3C protease was added per litre culture to remove the His_6_-SUMO or HIS tag while the sample eluent was dialysed overnight at 4 °C against wash buffer I. Next day, the dialysed sample was passed through a further Ni-Sepharose column to remove cleaved tags and/or any uncleaved fusion protein. Samples of the fractionated eluent were applied to 12% NuPage BIS-TRIS denaturing gels to assess sample purity and the purified protein was concentrated using VivaSpin 20 centrifugal concentrators with a molecular weight cut-off (MWCO) of 10 or 5 kDa (depending on size) and stored at −20 °C in small aliquots. For samples used for NMR assignments, NMR titrations, BLI and crystallography a further size exclusion chromatography step was included to remove residual high-molecular-weight aggregates. For these purposes either a Superdex200 (16/60) or a Superdex75 (16/60) prep grade column was equilibrated with gel filtration buffer (10 mM Tris-HCl, 1 M NaCl, 1 mM TCEP, pH 8) and 5 mL purified protein was injected. The fractions containing the protein were collected and concentrated using VivaSpin 20 with a MWCO of 10 or 5 kDa (depending on size).

To produce ^15^N- and ^13^C- labelled proteins for NMR assignment, cells were grown in minimal medium (0.3 mM CaCl_2_, 1 mM MgSO_4_, 1 µL mL^−1^ thiamine, 1 µL mL^−1^ d-biotin, 0.1 µL mL^−1^ ampicillin, trace elements and M9 salts) containing 1 g per litre ^15^N-ammonium sulphate and 2 g per litre ^13^C-glucose as sole sources of carbon and nitrogen.

To produce seleno-methionine (SeMet)-labelled N-terminal-RRM1 construct, cells were grown to an OD_600_ = 0.5 at 37 °C in M9 minimal medium. Then the culture was cooled to 20 °C. After 15 min, an amino-acid supplement (l-lysine, l-phenylalanine, l-threonine to a final concentration of 100 mg L^−1^, l-isoleucine, l-leucine, l-valine and l-SeMet to a final concentration of 40 mg L^−1^, respectively) was added to inhibit endogenous methionine biosynthesis and initiate SeMet incorporation. After a further 30 min protein expression was induced by the addition of 0.5 mM IPTG, and cells were grown for a further 18 h. Incorporation of selenomethionine was assessed by mass spectrometry, which showed that the incorporation was >95%.

### RNA sample preparation

Protected RNA oligonucleotides were purchased from GE Healthcare, Dharmacon, and deprotected as required using the standard protocol provided by the company. Ribo-oligonucleotides were: hEXO-motif: AGGCU, full-length miR-3470: UCACUUUGUAGACCAGGCUGG, 14mer miR-3470: GUAGACCAGGCUGG and chimera miR-3470: UCACUUUGUAGACCAAGUGUU.

### NMR spectroscopy

All NMR experiments were carried out using Bruker Avance NMR spectrometers operating at 600, 700, 800 and 950 MHz ^1^H frequencies, except for relaxation experiments on the dSyncrip protein, which were recorded on a Varian Inova spectrometer operating at 600 MHz ^1^H-frequency. Temperature was maintained at 298 K. Data were processed using the NMRPipe/Draw suite of programmes^38^ or Topspin. Assignment of protein resonances was performed in CARA^39^ and CCPNMR analysis^40^, titrations were analysed in Sparky^41^ and CCPNMR analysis.

### Protein backbone assignment experiments

Backbone resonances assignments for ^1^H, ^15^N, ^13^Cα and ^13^Cβ, and ^13^C′ chemical shifts of the dSyncrip N and NeR1 constructs were obtained from standard HNCACB, CBCA(CO)NH, HNCO, HNCA and HN(CA)CO experiments recorded on a 0.6 mM protein sample in 5 mM Tris-HCl, 50 mM NaCl, 1 mM TCEP-HCl, 0.04% NaN_3_ at pH 7.4. For hSyncrip NeR1 and eR1 the backbone assignments for ^1^H, ^15^N, ^13^Cα and ^13^Cβ chemical shifts were obtained from HNCA and CBCA(CO)NH experiments recorded on 0.6 mM protein samples in the buffer and conditions above. The ^1^H and ^15^N amide resonances of hSyncrip N-terminal domain and of the dSyncrip eR1 were assigned based on the superimposition of the spectra of the individual domains and the di-domains.

### NMR relaxation experiments and RDC measurements

^15^N T_1_ and T_2_ values and ^15^N heteronuclear NOE values were obtained from standard experiments^42^ recorded at 600 MHz ^1^H frequency on 0.3 mM samples of dSyncrip NeR1 and eR1 proteins. 10, 100, 200, 400, 500, 750, 1000, 1250, 1500 ms delays and 9, 18, 27, 36, 45, 63, 81, 100 ms delays were used, respectively, for T_1_ and T_2_ experiment recorded on dSyncrip NeR1. 10, 100, 200, 300, 400, 700, 800, 1000, 1500 ms delays and 8, 16, 24, 32, 40, 48, 72, 80, 96, 120, 144 ms delays were used, respectively, for T_1_ and T_2_ experiment recorded on dSyncrip eR1. Heteronuclear NOE values were obtained from standard experiments^42^.

RDCs were obtained for the dSyncrip NeR1 construct. In-Phase and Anti-Phase (IPAP) experiments were recorded on a 0.3 mM sample of ^15^N-labelled NeR1 in NMR buffer and filamentous phage Pf1 (from ASLA Biotech Ltd, Latvia). The RDC values were obtained by subtracting the reference value in isotropic solution. All data were processed with NMRPipe and the spectra were analysed in CCPNMR analysis. Fitting of the measured RDCs to those calculated from the crystal structure was performed using the program Module^43^ using either one tensor or two tensors corresponding to the two domains.

### NMR titration experiments

NMR titrations were performed in 5 mM Tris_HCl, 50 mM NaCl, 0.5 mM TCEP-HCl, at pH 7.4. buffer recording ^15^N SOFAST NMR spectra of protein–RNA complexes at the concentrations and ratios below. A 50 µM sample of dSyncrip N-terminal domain was titrated with four and five-nucleotide fully randomised oligonucleotide pool (4N and 5N RNAs) at 1:4 protein:RNA ratio. A 50 µM sample of hSyncrip N-terminal domain was titrated with 4 N RNA pool up to a 1:4 protein:RNA ratios. A 50 µM sample of hSyncrip N-terminal domain was also titrated with hEXO RNA at 0, 0.5, 1, 1.5, 2, 3, 4 and 6 protein:RNA ratio. Fifty micromolar samples of hSyn and dSyn extended RRM1 were titrated with NNNANN, NNNCNN, NNNGNN and NNNUNN RNA (for reasons of availability we used an oligo series) at 1:4 and 1:16 protein:RNA ratios. One-hundred and twenty micromolar samples of hSyn NeR1R2R3 were titrated with either hEXO (at 0, 0.5, 1 protein:RNA ratios) or full-length miR-3470 (at 0 and 1 protein:RNA ratios). Fifty micromolar samples of hSyncrip NeR1 wild-type and R60A G97L mutant were titrated with hEXO RNA at 0, 1 and 2 protein:RNA ratios.

SIA experiments^34^ were performed on 25 µM samples of human and Drosophila N-terminal proteins. Protein–RNA ratios employed were 1:4 and 1:8, respectively, to better match optimal shift changes. Experiments were performed as described^44^. Briefly, NMR data were acquired using a Bruker Avance III NMR spectrometer operating at 700 MHz and equipped with a 5 mm TCI cryoprobe. Samples were stored in 3 mm NMR tubes at 4 °C within a Bruker Samplejet auto-sampler and loaded automatically after a short pre-heating 25 °C step. Locking, tuning, matching and shimming were performed automatically.

### X-ray crystallography

Drosophila Syncrip NeR1 was crystallised using sitting drop vapour diffusion. Typically, a 5 mg mL^−1^ solution of NeR1 in in either Gel filtration buffer or NMR sample buffer was mixed in a 1:1 ratio with mother liquor. Initial conditions were optimised and drops were seeded to produce fewer, larger crystals. The best NeR1 crystals formed in 2.2 M ammonium sulphate, 0.1 M sodium citrate pH 6 with seeding and incubation at 18 °C for 5–7 days. Crystals were cryoprotected with 20% (v/v) glycerol and frozen by direct immersion in liquid nitrogen. The crystals belong to the space group P2_1_ with two copies of NeR1 present in the asymmetric unit (ASU). Seleno-methionine (Se Met) derived protein was crystallised under the same conditions. The diffraction quality of the tested crystals was highly variable, often being highly anisotropic in one direction.

Diffraction data sets were collected at 100 K at the I03 beamline at Diamond Light Source (Didcot, UK). The structure of NeR1 was solved by SAD using a fine-sliced data set (0.15°) of Se-Met derived protein recorded at 0.9795 Å. X-ray Detector Software (XDS)^45^ was used to process the data which extended to 3.1 Å but with anomalous signal only extending to 4.2 Å. Determination of the number of selenium atoms present in the ASU was performed using SAD methods in PHENIX^46^ and the initial map phased using ten Se sites. The backbone of the secondary structure elements of RNA15 (PDB code:  2X1B) was used as an initial model in the automated model-building function in PHENIX, which yielded a map-model CC of 0.67. Manual, real space building/re-building of the model was performed using COOT^47^.

The native data  were processed using XDS with data cut at 2.2 Å and a single chain from the SAD model was used to perform molecular replacement (MR) in PHASER^48^. Iterative rounds of reciprocal space and real space refinement in PHENIX or REFMAC^49^ and COOT were performed to complete the model. TLS groups were calculated using TLSMD^50^ and only used in the final round of refinement. The model was refined to a final *R*_work_/*R*_free_ of 0.174/0.206 and has good geometry as determined by PROCHECK^51^. Details of the crystal parameters and refinement statistics are presented in Supplementary Table 2, and the co-ordinates and structure factors have been deposited in the protein data bank (accession code: 6ES4). All structural images were generated using PyMol (The PyMOL Molecular Graphics System, Version 1.8 Schrödinger, LLC).

### HADDOCK

The HADDOCK runs were performed using the GURU interface of the WeNMR/West-Life GRID-enabled web portal for HADDOCK 2.2^31,32^ and standard parameters with the exception that only 5% of the input data were discarded. As experimental input parameters, all residues showing CSPs > 0.02 ppm were marked as active with HADDOCK determining the passive residues automatically. The RDC data were input with different tensor values for the NURR-domain (*D* −17.7 and *R* 0.22) and the extRRM1 domain (*D* −18.0 and *R* 0.31) as obtained from the best fit of the RDC data to the crystal structure with the program PALES^52^. Two-hundred structures were calculated that grouped into three clusters (Clusters 1,2,3 having 106, 68, 21 structures respectively) representing 97.5% of the structures. Cluster 1 is by far the largest and also has the lowest overall Haddock score (−71.4 +/− 3.2), with cluster 2 having a less favourable Haddock score (−66.8 +/− 1.0) and cluster 3 considerably lower score (−50.9 +/− 2.5). The results compare well with the tutorial example given in ref. ^53^.

### Biolayer interferometry measurements

BLI experiments were performed in 5 mM Tris-HCl, 50 mM NaCl, 1 mM TCEP, 0.5 mg mL^−1^ bovine serum albumin (BSA) pH 7.4 on an Octet Red 96 instrument (ForteBio, Inc. Menlo Park, CA) operating at 30 °C. 5′-Biotinylated miR-3470 RNA variations (14mer, full-length and chimera) (1 ng µL^−1^ solutions) were immobilised on streptavidin-coated biosensors and incubated with varying concentrations of hSyncrip NURR (2 × dilution series, 400–6.25 µM) and Syncrip NeR1R2R3 (2 × dilution series, 50–0.78 nM). Dissociation constants for RNA–protein interactions were determined from plots of the increase in BLI signal as a function of the protein concentration and fitting using non-linear regression using in-house software, as described^54^.

### Sequence alignments and modelling

All sequence alignments were carried out using T-Coffee multiple sequence alignment program^55^(accessible at http://www.ebi.ac.uk/Tools/msa/tcoffee/). All the alignment figures were generated by using Jalview/ClustalX^56^. The models of the human eRRM1 and NeR1 proteins were built using the SWISS-PROT modelling server^57^, with default parameters and the corresponding region of the two-domain Drosophila protein as an input template.

### UV-crosslinking RIP

RIP experiments were performed as in Santangelo et al.^10^, with the changes described below. A total volume of extract containing 3 mg of protein was precleared with protein G Dynabeads (Invitrogen, San Diego, CA, USA) for 30 min at 4 °C with end-over end rotation and then incubated with 5 µg mouse anti-hnRNPQ antibody (05–1517 EMD Millipore, Merck SpA, Germany) or mock antibody (12,371 Mouse IgG, EMD Millipore, Merck SpA, Germany) for 16 h at 4 °C with end-over end rotation. Twenty microlitres of Protein G Dynabeads (Invitrogen, San Diego, CA, USA), blocked with 3% BSA, were added for 4 h, followed by five washes with denaturing wash buffer^58^. In brief, co-immunoprecipitated miRNAs were extracted using Total RNA miniKIT (geneAID, Taiwan) reverse transcribed and analysed by quantitative PCR (qPCR). miRNA fold enrichment in immunoprecipitated samples was expressed as fold with respect to IgG isotypic control.

### Data availability

Structure Factors and co-ordinates for Syncrip NeR1 have been deposited in the protein data bank with accession number 6ES4. All relevant data are available from the corresponding authors upon reasonable request.

## Electronic supplementary material

Supplementary Information
