# Supplementary material for: A cryptic RNA-binding domain mediates Syncrip recognition and exosomal partitioning of miRNA targets
Source: Nat Commun. 2018 Feb 26;9:831. doi: 10.1038/s41467-018-03182-3 (PMC5827114; doi:10.1038/s41467-018-03182-3)
Supplement: Supplementary file 1 — Supplementary Information [file 41467_2018_3182_MOESM1_ESM.pdf]

A CRYPTIC RNA-BINDING DOMAIN MEDIATES SYNCRIP RECOGNITION AND EXOSOMAL PARTITIONING OF miRNA TARGETS

Hobor et al.

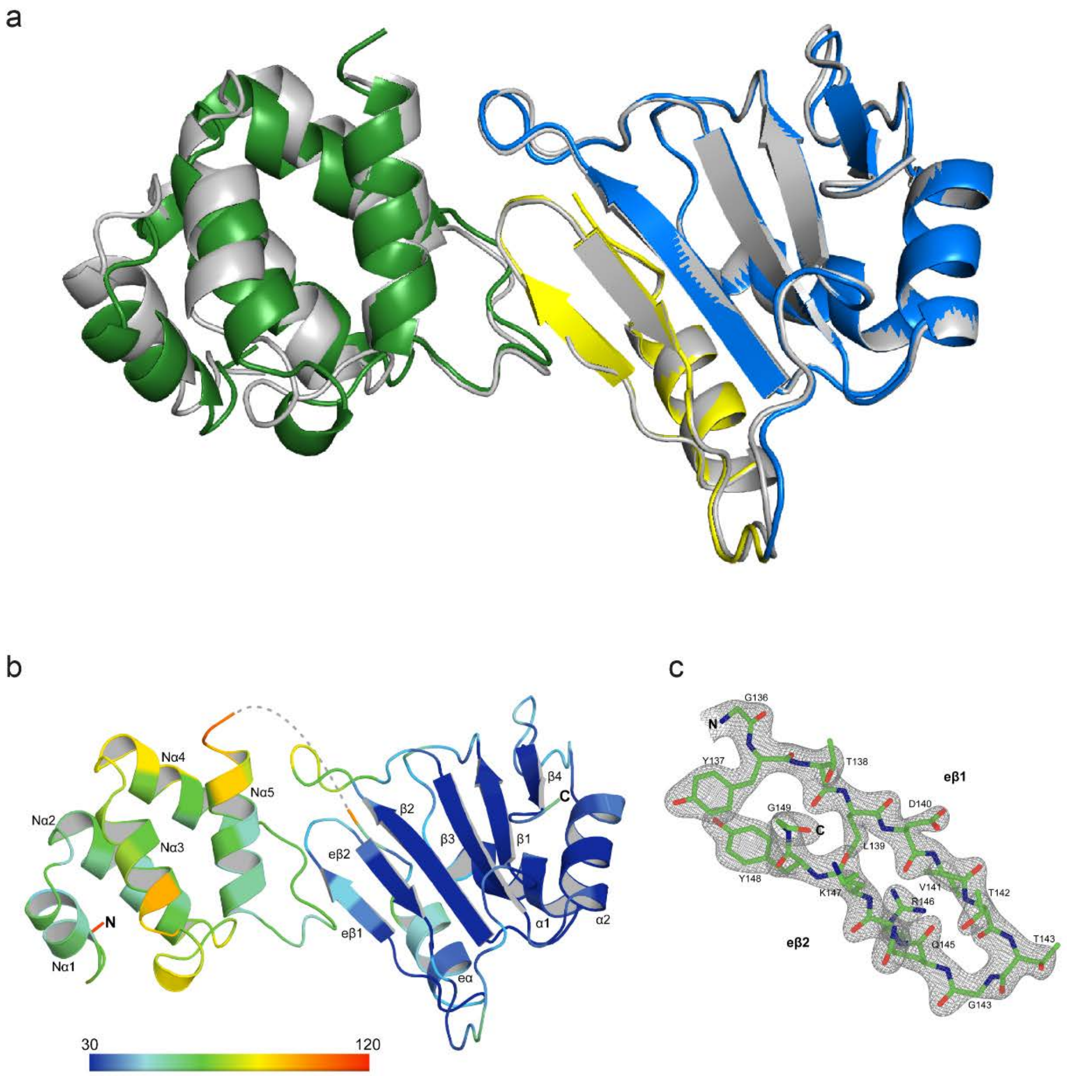

Supplementary Figure 1: Structure of Syncrip N-terminal and extended RRM1 domains.

(a) Structural superimposition of the two Syncrip NeR1 copies within a unit cell. Structure A is depicted with the same colour coding as Fig. 2 and structure B is shown in grey. Some minor differences exist in the orientation of the secondary structures of the N-terminal domain, but not in the interface between the two domains.

(b) Mapping of the crystallographic B factors on the cartoon representation of the structure. The colour scale goes from low (blue) to high (red). Residues in the N-terminal domains have significant higher B factors.

(c) Fitting of sidechains into the 2.2 Å electron density map, contoured at 2.5 sigma

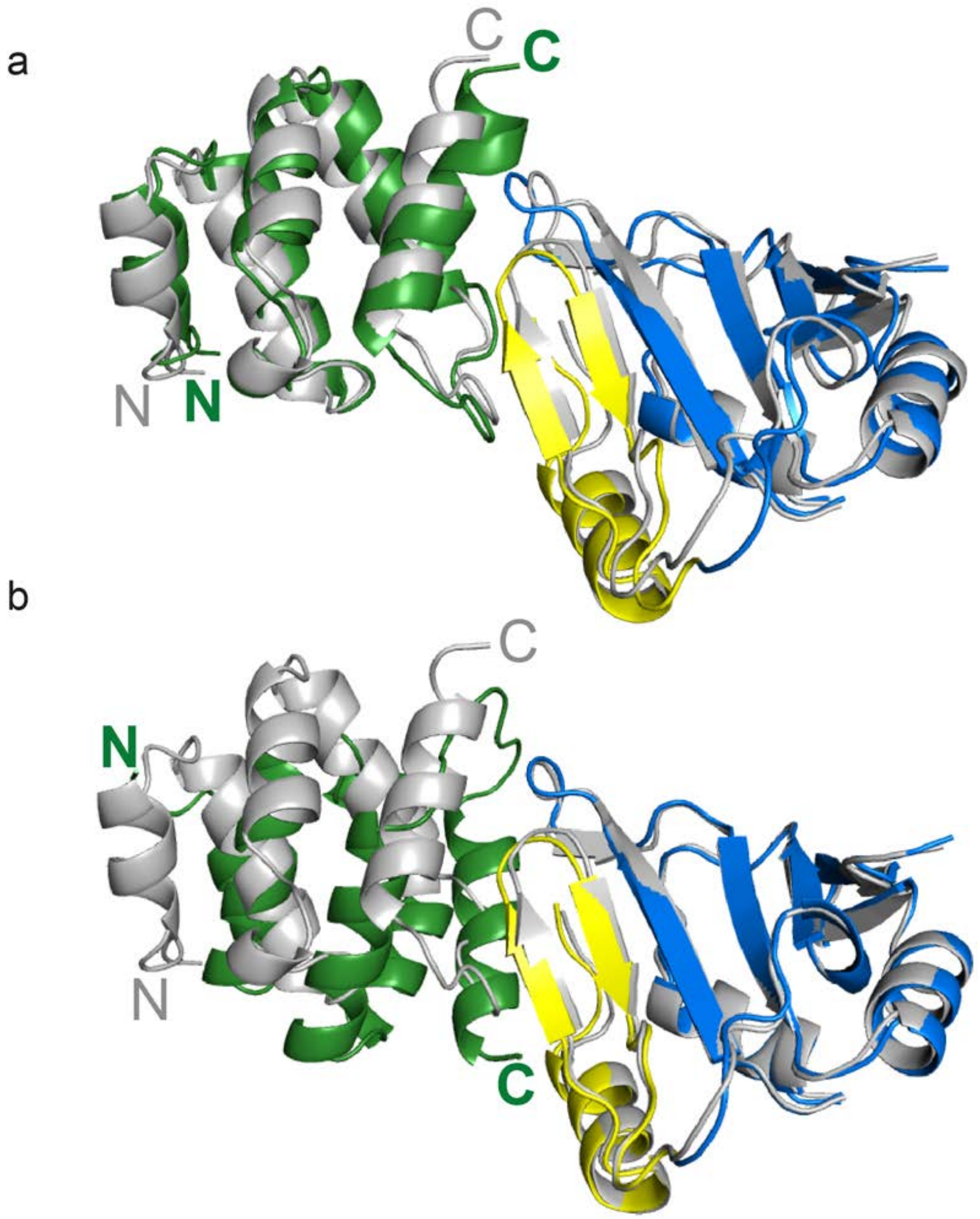

Supplementary Figure 2: Cartoon representation of the two main families from a CSP and RDC-driven HADDOCK docking of the NURR and eRRM1 domains.

(**a**) Structural superimposition of the Drosophila crystal structure (grey, this study) and the best model from the lower energy more populated (105 over 199 models) HADDOCK cluster (domain colour-coded), shown as cartoon representations.

(**b**) Corresponding structural superimposition with the best model of the second more populated cluster (67 over 199 models). The relative orientation of the two domains is ~180 degrees rotated.

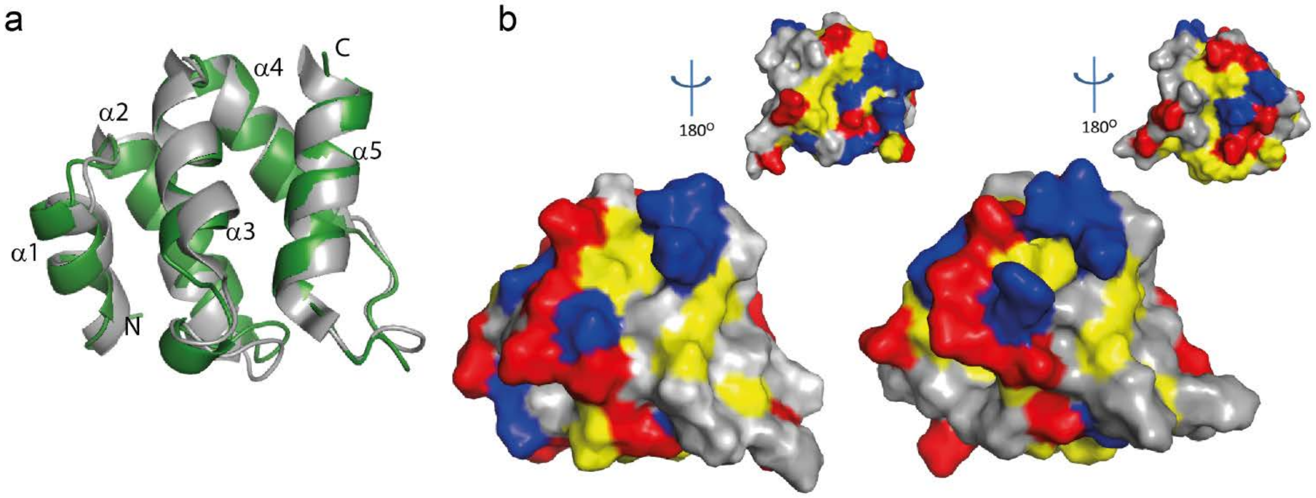

Supplementary Figure 3: Structure of Drosophila and human N-terminal 'acidic' domain

(a) Structural superimposition of the human (grey, PDB: 2MXT) and Drosophila N-terminal 'acidic' domains, shown in cartoon representation.

(b) Hydrophobicity and charge distribution on the Drosophila (left) and human (right) N-terminal domains surfaces. Negatively charged residues are coloured in red, positively charged residues in blue and hydrophobic residues in yellow.

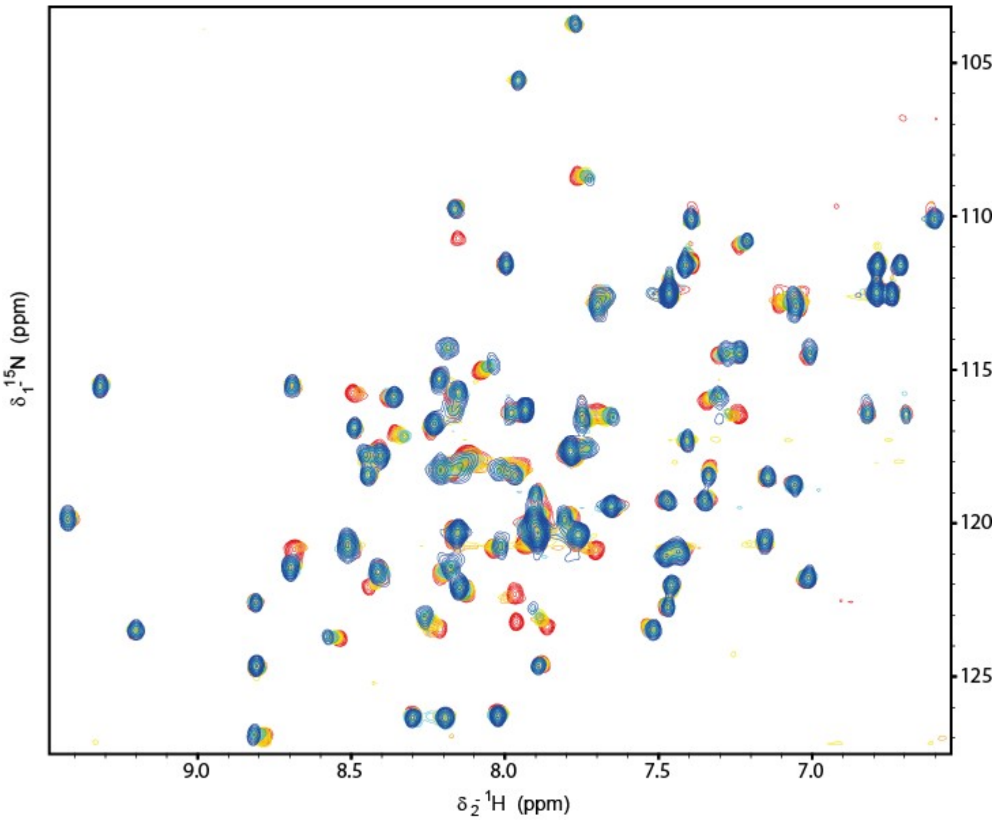

Supplementary Figure 4: Titration of human Syncrip NURR domain with hEXO RNA.

Superimposition of the ^{15}N-correlation spectra of the NURR domain of human Syncrip at different ratios of AGGCU RNA. Spectra are colour coded as red, orange, yellow, green, cyan, blue, purple, magenta for 0, 0.5, 1, 1.5, 2, 3, 4, 6 protein:RNA ratios respectively.

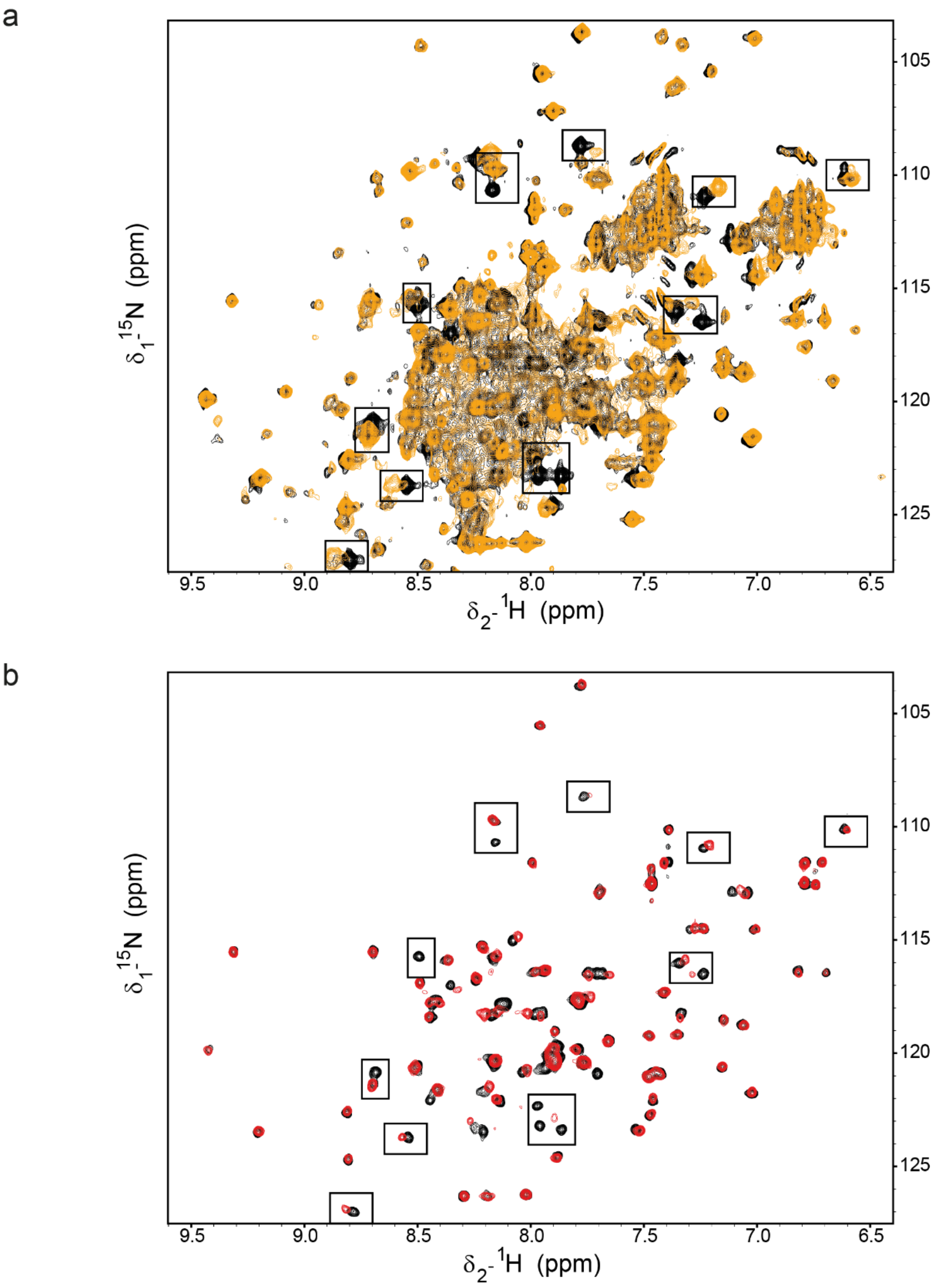

Supplementary Figure 5. Titration of human Syncrip four-domain NeR1R2R3 construct with hEXO RNA.

(a) Binding of the NURR domain to the AGGCU hEXO-motif monitored by NMR. Superimposition of the free (black) and RNA-bound (1:1 ratio, red) protein spectra, as in Figure7. Resonances in the well-dispersed region of the spectrum that change significantly upon binding are boxed.

(b) Binding of the four-domain NeR1R2R3 construct to the AGGCU hEXO motif. Superimposition of the free (black) and RNA-bound (1:1 ratio, orange) protein spectra, as in Figure 7. Boxed resonances are as in panel (a).

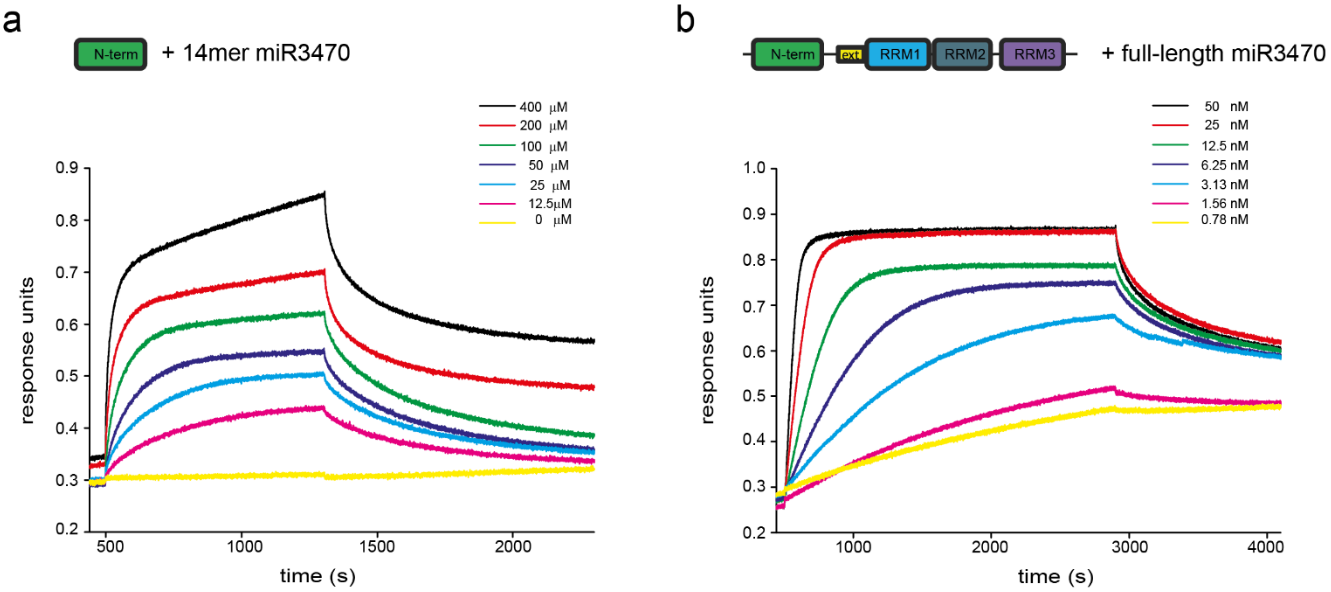

Supplementary Figure 6. Binding of Syncrip constructs to the target miR-3470.

(a) BLI response of Streptavidin-coated sensors coated with biotinylated 14mer miR-3470 RNA and then exposed to increasing concentrations of the NURR domain. Data are aligned using the response in buffer solution. The baseline, association, and dissociation steps are displayed.

(b) BLI response of Streptavidin-coated sensors coated with biotinylated full-length miR-3470 RNA and exposed to increasing concentrations of NeR1R2R3. Data are aligned using the response in the buffer solution. The baseline, association, and dissociation steps are displayed.

ENSG0000 O60506	SYNCRIP	AIEALKEFNEDGALAVLQQFK	61	81	RBDpep	UniqueGene	other	LysC	VAEKLDEIYVAGLVAHSDLDERAIEALK	39	81
ENSG0000 O60506	SYNCRIP	EFNEDGALAVLQQFK	67	81	RBDpep	UniqueGene	other	ArgC	AIEALKEFNEDGALAVLQQFKDSDLSH	61	103
ENSG0000 O60506	SYNCRIP	KYGGPPPDSVYSGQQPSVGTEIFVGI	143	168	RBDpep	UniqueGene	classical	LysC	IKALLERTGYTLDVTTGQRKYGGPPPDS	124	168
ENSG0000 O60506	SYNCRIP	YGGPPPDSVYSGQQPSVGTEIFVGK	144	168	RBDpep	UniqueGene	classical	ArgC	KYGGPPPDSVYSGQQPSVGTEIFVGKII	143	171
ENSG0000 O60506	SYNCRIP	YGGPPPDSVYSGQQPSVGTEIFVGK	144	168	RBDpep	UniqueGene	classical	LysC	YGGPPPDSVYSGQQPSVGTEIFVGK	144	168
ENSG0000 O60506	SYNCRIP	DLFEDELVPLFEK	172	184	RBDpep	UniqueGene	classical	LysC	IPRDLFEDELVPLFEK	169	184
ENSG0000 O60506	SYNCRIP	GYAFVTFCTK	204	213	RBDpep	UniqueGene	classical	LysC	AGPIWDLRLMMDPLTGLNRGYAFVTF	185	213
ENSG0000 O60506	SYNCRIP	LMMDPLTGLNR	193	203	RBDpep	UniqueGene	classical	LysC	AGPIWDLRLMMDPLTGLNRGYAFVTF	185	213
ENSG0000 O60506	SYNCRIP	TKEQILEEFSK	255	265	RBDpep	UniqueGene	classical	LysC	SKTKEQILEEFSK	253	265
ENSG0000 O60506	SYNCRIP	NLANTVTEEILEK	344	356	RBDpep	UniqueGene	classical	LysC	VKVLFVRNLANTVTEEILEK	337	356
ENSG0000 O60506	SYNCRIP	LKDYAFIHFDER	370	381	RBDpep	UniqueGene	classical	ArgC	VKKLKDYAFIHFDER	367	381
ENSG0000 O60506	SYNCRIP	DYAFIHFDER	372	381	RBDpep	UniqueGene	classical	LysC	LKDYAFIHFDERDGAVK	370	386
ENSG0000 O60506	SYNCRIP	LKDYAFIHFDER	370	381	RBDpep	UniqueGene	classical	LysC	LKDYAFIHFDERDGAVK	370	386
ENSG0000 O60506	SYNCRIP	LDEIYVAGLVAHSDLDER	43	60	CandidateR	UniqueGene	other	ArgC	MATEHVNGNGTEEPMDTTSAVIHSEN	1	60
ENSG0000 O60506	SYNCRIP	VAEKLDEIYVAGLVAHSDLDER	39	60	CandidateR	UniqueGene	other	ArgC	MATEHVNGNGTEEPMDTTSAVIHSEN	1	60
ENSG0000 O60506	SYNCRIP	LDEIYVAGLVAHSDLDER	43	60	CandidateR	UniqueGene	other	LysC	VAEKLDEIYVAGLVAHSDLDERAIEALK	39	66
ENSG0000 O60506	SYNCRIP	VAEKLDEIYVAGLVAHSDLDER	39	60	CandidateR	UniqueGene	other	LysC	VAEKLDEIYVAGLVAHSDLDERAIEALK	39	66
ENSG0000 O60506	SYNCRIP	EFNEDGALAVLQQFK	67	81	CandidateR	UniqueGene	other	LysC	EFNEDGALAVLQQFK	67	81
ENSG0000 O60506	SYNCRIP	KYGGPPPDSVYSGQQPSVGTEIFVGI	143	168	CandidateR	UniqueGene	classical	ArgC	KYGGPPPDSVYSGQQPSVGTEIFVGKII	143	171
ENSG0000 O60506	SYNCRIP	LMMDPLTGLNR	193	203	CandidateR	UniqueGene	classical	ArgC	LMMDPLTGLNR	193	203
ENSG0000 O60506	SYNCRIP	GYAFVTFCTK	204	213	CandidateR	UniqueGene	classical	ArgC	GYAFVTFCTKEAAQEAVKLYNNHEIR	204	229
ENSG0000 O60506	SYNCRIP	EQILEEFSK	257	265	CandidateR	UniqueGene	classical	ArgC	LFVGSIPKSKTKEQILEEFSKVTEGLTDV	245	286
ENSG0000 O60506	SYNCRIP	AFSQFGK	357	363	CandidateR	UniqueGene	classical	ArgC	NLANTVTEEILEKAFSQFGKLER	344	366
ENSG0000 O60506	SYNCRIP	AFSQFGKLER	357	366	CandidateR	UniqueGene	classical	ArgC	NLANTVTEEILEKAFSQFGKLER	344	366
ENSG0000 O60506	SYNCRIP	DYAFIHFDER	372	381	CandidateR	UniqueGene	classical	ArgC	VKKLKDYAFIHFDER	367	381
ENSG0000 O60506	SYNCRIP	AMEEMNGKDLEGENIEIVFAKPPDC	387	412	CandidateR	UniqueGene	classical	LysC	AMEEMNGKDLEGENIEIVFAKPPDQKI	387	414
ENSG0000 O60506	SYNCRIP	DLFEDELVPLFEK	172	184	Input	UniqueGene	classical	ArgC	DLFEDELVPLFEKAGPIWDLR	172	192
ENSG0000 O60506	SYNCRIP	TKEQILEEFSK	255	265	Input	UniqueGene	classical	ArgC	LFVGSIPKSKTKEQILEEFSKVTEGLTDV	245	286
ENSG0000 O60506	SYNCRIP	VTEGLTDVILYHQPDDK	266	282	Input	UniqueGene	classical	ArgC	LFVGSIPKSKTKEQILEEFSKVTEGLTDV	245	286
ENSG0000 O60506	SYNCRIP	EQILEEFSK	257	265	Input	UniqueGene	classical	LysC	SKTKEQILEEFSK	253	265
ENSG0000 O60506	SYNCRIP	VTEGLTDVILYHQPDDK	266	282	Input	UniqueGene	classical	LysC	VTEGLTDVILYHQPDDK	266	282
ENSG0000 O60506	SYNCRIP	VTEGLTDVILYHQPDDKK	266	283	Input	UniqueGene	classical	LysC	VTEGLTDVILYHQPDDKKKNRGFCFLEY	266	297
ENSG0000 O60506	SYNCRIP	VWGNVGTVEWADPIEDPDPEVMA	313	336	Input	UniqueGene	classical	ArgC	RRLMSGKVKVWGNVGTVEWADPIED	304	343
ENSG0000 O60506	SYNCRIP	VWGNVGTVEWADPIEDPDPEVMA	313	336	Input	UniqueGene	other	LysC	VKVWGNVGTVEWADPIEDPDPEVMA	311	336
ENSG0000 O60506	SYNCRIP	NLANTVTEEILEK	344	356	Input	UniqueGene	classical	ArgC	NLANTVTEEILEKAFSQFGKLER	344	366
ENSG0000 O60506	SYNCRIP	AFSQFGK	357	363	Input	UniqueGene	classical	LysC	AFSQFGK	357	363
ENSG0000 O60506	SYNCRIP	DLEGENIEIVFAKPPDQK	395	412	Input	UniqueGene	classical	ArgC	DGAVKAMEEMNGKDLEGENIEIVFAK	382	413
ENSG0000 O60506	SYNCRIP	DLEGENIEIVFAKPPDQK	395	412	Input	UniqueGene	classical	LysC	DLEGENIEIVFAKPPDQKRK	395	414
ENSG0000 O60506	SYNCRIP	QTNNQNWGSQPIAQQPLQGGDHS	579	607	Input	UniqueGene	other	LysC	RRQTNNQNWGSQPIAQQPLQGGDHS	577	607
ENSG0000 O60506	SYNCRIP	SENQEFYQDTFGQQWK	608	623	Input	UniqueGene	other	ArgC	RQTNNQNWGSQPIAQQPLQGGDHS	578	623
ENSG0000 O60506	SYNCRIP	SENQEFYQDTFGQQWK	608	623	Input	UniqueGene	other	LysC	SENQEFYQDTFGQQWK	608	623

Supplementary Table 1. RNA binding sites in Syncrip identified by RBDmapBinding of Syncrip constructs to the target miR-3470.

The table reports the peptides mapping to Syncrip extracted from the RBDmap analysis of HeLa RBPs. Peptides are classified in three groups: RBDpeps (red) and candidate RBDpeps (salmon) refer to RNA-binding sites assigned with FDR<0.01 or FDR<0.1, respectively. Released peptides (cyan) are enriched in the supernatant of the second oligo(dT) capture after proteolytic treatment (Fig. 1b); thus lacking RNA binding. The MS-identified peptide corresponds to the tryptic moiety directly identified by mass spectrometry. The ArgC/LysC fragments are generated by extension of the MS-identified peptides to the closest LysC or ArgC cleavage sites to recapitulate the larger peptide crosslinked to RNA after the treatment with LysC or ArgC (Fig. 1b).

**Data Collection**	**Syp28 (Se-Met)**	**Syp28**
Space group	$P2_1$	$P2_1$
Cell dimensions		
a, b, c (Å)	34.14, 128.33, 81.58	33.78, 128.53, 79.58
α, β, γ (◦)	90, 100.03, 90	90, 101.12, 90
Wavelength	0.9795	0.9795
Resolution (Å)	50.0-3.1 (3.27-3.1)*	50-2.2 (2.33-2.20)
No. reflections	43127 (6581)	112896 (16903)
R_{meas}	9.1 (43.9)	4.5 (42.1)
I / σI	9.35 (2.12)	19.15 (2.95)
$CC_{1/2}$	0.995 (0.822)	0.999 (0.868)
Completeness (%)	94.1 (90.4)	99.0 (96.8)
Multiplicity	1.84 (1.83)	3.4 (3.1)
Anom multiplicity	1.83	
FOM (Phenix)	0.303	
**Refinement**		
Resolution (Å)		37.6 – 2.2
R_{work}/R_{free}		17.4/20.6
No. atoms		
Total (residues)		3279 (3144)
Water		76
B-factors		
Wilson		44.1
Average		60.0
R.m.s. deviations		
Bond lengths (Å)		0.003
Bond angles (◦)		0.75

*Values in parenthesis refer to the highest resolution shell

Supplementary Table 2. X-ray data collection and refinement statistics.

```
   1 atggcaacgg aacacgtcaa cggcaatggc acggaagaac cgatggatac gacctcagca
  61 gtgattcata gcgaaaactt tcaaacgctg ctggatgcag gtctgccgca gaaagtggct
 121 gaaaaactgg acgaaattta tgtggcgggc ctggttgccc atagtgatct ggacgaacgt
 181 gcgatcgaag ccctgaaaga atttaacgaa gatggtgcac tggctgtcct gcagcaattc
 241 aaagatagtg acctgtccca cgttcagaat aaatccgcct ttctgtgcgg cgtcatgaaa
 301 acctatcgcc aacgtgaaaa acagggtacg aaagttgcag atagctctaa aggcccggac
 361 gaagcaaaaa ttaaagctct gctggaacgt accggttaca cgctggatgt taccaccggt
 421 cagcgtaaat atggtggccc gccgccggac tcagtctaca gcggtcagca accgagcgtt
 481 ggtaccgaaa tttttgtcgg caaaatcccg cgcgacctgt ttgaagacga actggtgccg
 541 ctgttcgaaa aagcaggccc gatttgggat ctgcgtctga tgatggaccc gctgaccggt
 601 ctgaaccgcg gttatgcctt tgtcaccttc tgcacgaaag aagcggccca ggaagcagtg
 661 aaactgtaca acaatcatga aatccgttca ggcaaacaca ttggtgtctg tatctcggtg
 721 gcgaacaatc gcctgtttgt tggtagcatt ccgaaatcta aaaccaaaga acaaatcctg
 781 gaagaatttt caaaagtgac cgaaggtctg acggatgtta ttctgtatca tcagccggat
 841 gacaaaaaga aaaaccgtgg cttttgtttc ctggaatacg aagatcacaa aaccgcagcg
 901 caggcccgtc gccgtctgat gagcggtaaa gtgaaagttt ggggcaatgt tggtacggtc
 961 gaatgggccg atccgatcga agatccggac ccggaagtga tggcaaaagt caaagtgctg
1021 tttgtccgca acctggctaa taccgtgacg gaagaaattc tggaaaaagc gtttagccag
1081 ttcggtaaac tggaacgtgt gaaaaaactg aaagattacg ccttcatcca tttcgatgaa
1141 cgcgacggcg cagttaaagc tatggaagaa atgaacggca aagatctgga aggtgaaaat
1201 attgaaatcg tgttcgctaa accgccggat caaaaacgta aagaacgcaa agcgcaacgt
1261 caggcggcca aaaaccagat gtacgatgac tactactact acggtccgcc gcatatgccg
1321 ccgccaaccc gtggtcgtgg tcgcggcggt cgtggcggtt atggttaccc gccggattat
1381 tacggctacg aagattacta cgactactac ggttacgact accacaatta tcgcggcggt
1441 tacgaagatc cgtactatgg ctacgaagat ttccaagtgg gtgcccgcgg ccgcggcggt
1501 cgtggcgccc gcggtgcagc tccgagccgt ggtcgcggtg cggccccgcc gcgtggtcgt
1561 gccggttata gtcagcgtgg cggtccgggc tccgcacgtg gcgttcgcgg tgcgcgtggc
1621 ggtgcccagc aacagcgtgg ccgcggtgtg cgcggtgctc gtggcggtcg cggcggtaat
1681 gttggcggta aacgcaaagc ggatggctat aatcagccgg acagcaaacg ccgtcaaacc
1741 aacaatcaga actggggttc tcaaccgatt gcgcaacagc cgctgcaggg cggtgatcac
1801 tctggcaatt atggctacaa atcggaaaac caagaatttt atcaagacac cttcggtcag
1861 cagtggaaat ga
```

Supplementary Table 3. Codon optimised sequence of hnRNP-Q (full-length protein).

Primer (aa pos)	Sequence
Dm 16FWD	CAGGGACCCGGTGCCGATGACCGTGGCGATGGC
Dm 118FWD	CAGGGACCCGGTGCAACTGTCAAAGGTCCCGAC
Dm 243 REV	GGCACCAGAGCGTTACGGTACGCTTATATTTATTTTTAGACACTTGCCGGGTTTTATTTCG
Hs 16 FWD	CAGGGACCCGGTGATACGACCTCAGCAGTGATT
Hs 118FWD	CAGGGACCCGGTGGCCCGGACGAAGCAAAAATT
Hs 109 REV	GGCACCAGAGCGTTAACCCTGTTTTTCACGTTGGCG
Hs 242 REV	GGCACCAGAGCGTTAGTTCGCCACCGAGATACAGAC
Hs 428 REV	GGCACCAGAGCGTTAGTACATCTGGTTTTTGGCCGC
R60AG97L FWD1	GCTTCGATCGCAGCTTCGTCCAGATCACTATGGG
R60AG97L REV1	CCCATAGTGATCTGGACGAAGCTGCGATCGAAGC
R60AG97L FWD2	GTTGGCGATAGGTTTTCATGACTAGGCACAGAAAGGCGGATTTATTC
R60AG97L REV2	GAATAAATCCGCCTTTCTGTGCCTAGTCATGAAAACCTATCGCCAAC

Supplementary Table 4. Primers used in the study.
